# Temporal Dynamics of DNA Methylation Patterns in Response to Rearing Juvenile Steelhead (*Oncorhynchus mykiss*) in a Hatchery versus Simulated Stream Environment

**DOI:** 10.3390/genes10050356

**Published:** 2019-05-09

**Authors:** Mackenzie R. Gavery, Krista M. Nichols, Barry A. Berejikian, Christopher P. Tatara, Giles W. Goetz, Jon T. Dickey, Donald M. Van Doornik, Penny Swanson

**Affiliations:** 1 University of Washington, School of Aquatic and Fishery Sciences, 1122 NE Boat St., Seattle, WA 98105, USA; Giles.Goetz@noaa.gov (G.W.G.); Jon.Dickey@noaa.gov (J.T.D.); 2 Conservation Biology Division, Northwest Fisheries Science Center, National Marine Fisheries Service, NOAA, 2725 Montlake Blvd. E., Seattle, WA 98112, USA; Krista.Nichols@noaa.gov; 3 Environmental and Fisheries Sciences Division, Northwest Fisheries Science Center, National Marine Fisheries Service, NOAA, 7305 Beach Dr. East, Port Orchard, WA 98366, USA; Barry.Berejikian@noaa.gov (B.A.B.); Chris.P.Tatara@noaa.gov (C.P.T.); 4 Conservation Biology Division, Northwest Fisheries Science Center, National Marine Fisheries Service, NOAA, 7305 Beach Dr. East, Port Orchard, WA 98366, USA; Don.Vandoornik@noaa.gov; 5 Environmental and Fisheries Sciences Division, Northwest Fisheries Science Center, National Marine Fisheries Service, NOAA, 2725 Montlake Blvd. E., Seattle, WA 98112, USA; Penny.Swanson@noaa.gov

**Keywords:** DNA methylation, epigenetics, steelhead, hatchery

## Abstract

Genetic selection is often implicated as the underlying cause of heritable phenotypic differences between hatchery and wild populations of steelhead trout (*Oncorhynchus mykiss*) that also differ in lifetime fitness. Developmental plasticity, which can also affect fitness, may be mediated by epigenetic mechanisms such as DNA methylation. Our previous study identified significant differences in DNA methylation between adult hatchery- and natural-origin steelhead from the same population that could not be distinguished by DNA sequence variation. In the current study, we tested whether hatchery-rearing conditions can influence patterns of DNA methylation in steelhead with known genetic backgrounds, and assessed the stability of these changes over time. Eyed-embryos from 22 families of Methow River steelhead were split across traditional hatchery tanks or a simulated stream-rearing environment for 8 months, followed by a second year in a common hatchery tank environment. Family assignments were made using a genetic parentage analysis to account for relatedness among individuals. DNA methylation patterns were examined in the liver, a relatively homogeneous organ that regulates metabolic processes and somatic growth, of juveniles at two time points: after eight months of rearing in either a tank or stream environment and after a subsequent year of rearing in a common tank environment. Further, we analyzed DNA methylation in the sperm of mature 2-year-old males from the earlier described treatments to assess the potential of environmentally-induced changes to be passed to offspring. Hepatic DNA methylation changes in response to hatchery versus stream-rearing in yearling fish were substantial, but few persisted after a second year in the tank environment. However, the early rearing environment appeared to affect how fish responded to developmental and environmental signals during the second year since novel DNA methylation differences were identified in the livers of hatchery versus stream-reared fish after a year of common tank rearing. Furthermore, we found profound differences in DNA methylation due to age, irrespective of rearing treatment. This could be due to smoltification associated changes in liver physiology after the second year of rearing. Although few rearing-treatment effects were observed in the sperm methylome, strong family effects were observed. These data suggest limited potential for intergenerational changes, but highlight the importance of understanding the effects of kinship among studied individuals in order to properly analyze and interpret DNA methylation data in natural populations. Our work is the first to study family effects and temporal dynamics of DNA methylation patterns in response to hatchery-rearing.

## 1. Introduction

Captive breeding programs are often used for the enhancement or to prevent extinction of species of conservation concern. Frequently, however, this approach may result in unintended phenotypic consequences that impact fitness [1]. For Pacific salmon and steelhead (*Oncorhynchus mykiss*), conservation hatcheries are one of the most commonly used management strategies to mitigate the dramatic declines of many populations on the West Coast of North America. Conservation hatcheries intend to supplement wild populations with fish that are genetically and phenotypically similar to the wild stocks, but data suggests that salmon and steelhead reared in hatcheries are phenotypically different than wild fish [2,3]. A number of studies have reported that hatchery fish exhibit a reduced reproductive success compared to wild fish when spawning in the wild [4,5] and that fitness loss can be observed within one or two generations in the hatchery [6,7].

It is now understood that the environment encountered during early development can have lasting effects on metabolic, growth, behavioral and life-history related traits in animals [8,9,10]. This is referred to as developmental plasticity, and is the concept that the same genotype can produce different phenotypes depending on extrinsic, environmental inputs during development [11]. Developmental plasticity can be beneficial for organisms by providing cues of the future environment that will be encountered, however, there is the potential for a phenotypic mismatch if the environment encountered during development is not predictive of the future environment [8]. This may be particularly important for developing hatchery-reared fish where environmental conditions, such as temperature, diet, seasonal food availability and photoperiod are manipulated to accelerate growth, but are likely not predictive of the future environment, once fish are released into the wild. These manipulations can result in significant differences in life history patterns between hatchery and natural-origin fish [12].

In fish, traits such as growth, sex differentiation, life history and behavioral performances can be influenced by the environmental conditions encountered during development [9]. Many of these studies have direct relevance to hatchery-rearing conditions. For example, it has recently been shown that rearing temperature has a significant effect on return date in Atlantic salmon (*Salmo salar*), with fish reared at 3 °C above normal temperature returning two weeks later on average than those reared at a normal temperature [13]. While observations of developmental plasticity are well-documented, the mechanisms underlying these phenotypes, which are likely to be epigenetic in nature, have remained relatively unstudied until recently when genomic tools have become available to study these mechanisms in non-model species [14,15,16].

Epigenetics refers to mechanisms that alter gene activity without modifications to the underlying DNA sequence [17]. Epigenetic mechanisms, including DNA methylation, histone modifications and non-coding RNAs, are important mediators of environmental plasticity, and unlike the DNA, epigenetic regulators can be directly influenced by the environment [18]. The role of epigenetics specifically in developmental plasticity has emerged mainly from studies in mammals [19,20], but increasing evidence is supporting the role of epigenetics in regulating the developmental plasticity in fish. For example, in European sea bass, which exhibits temperature dependent sex determination, exposure to high temperature in early development is associated with increased methylation of the aromatase (*cyp19a*) promoter and subsequent predominance of the male phenotype [21]. In rainbow trout, transcriptomic analyses suggest that epigenetic mechanisms may be involved in mediating growth and feed intake phenotypes observed in fish that had been nutritionally programmed to a plant-based diet at first feeding [22,23]. In Atlantic salmon, increased temperature during early development is associated with DNA methylation and gene expression changes in *myogenin*, a gene with important functions in muscle development [24]. Early life-stress is also associated with alterations in DNA methylation, gene expression and subsequent growth in Atlantic salmon [25].

The ability of early environmental information to be “stored” in the form of an epigenetic memory has also been explored recently in hatchery-reared salmonids. Two recent studies examined the potential for DNA methylation to store epigenetic information acquired in the hatchery-rearing environment as a first step toward understanding how epigenetic mechanisms may be contributing to loss of fitness phenotypes observed in hatchery-reared salmonids. Le Luyer et al. [26] examined genetic and epigenetic differentiation between hatchery- and natural-origin juvenile coho salmon (*Oncorhynchus kisutch*) from two independent rivers shortly after release from the hatchery. While genetic differentiation between hatchery- and natural-origin fish within a river was nonsignificant, substantial differences in DNA methylation in the muscle of juvenile hatchery and natural-origin coho were observed [26]. These results suggest that hatchery-rearing does indeed leave a DNA methylation “signal” on juvenile fish. However, environmentally-induced DNA methylation patterns may also be dynamic [27,28,29] and it is unclear whether differences in DNA methylation observed in juveniles are persistent, and thus have the potential to affect phenotypes in the adults, or reversible and DNA methylation patterns become indistinguishable after some period of time in the wild. Gavery et al. [30] addressed this question by analyzing genetic and epigenetic variation between hatchery- and natural-origin adult steelhead returning to the Methow River in Washington, USA. The results in adult steelhead support the findings of Le Luyer et al. [26], in that nonsignificant genetic (DNA sequence) variation was observed, but differences in DNA methylation were observed in both red blood cells (RBCs) and sperm, suggesting at least some of the environmentally-induced changes resulting from hatchery-rearing may be persistent. However, a high degree of variation in DNA methylation was observed within the hatchery- and natural- origin fish indicating that other factors such as genetic background and age could be contributing to the overall variation in DNA methylation [30]. While both of these studies are important first steps toward understanding the role of DNA methylation in mediating loss of fitness phenotypes observed in hatchery-reared Pacific salmon and steelhead, the results raise important questions about the plasticity or persistence of DNA methylation changes induced by the early rearing environment and highlight a lack of understanding of the sources of variation in DNA methylation in natural populations. Further, both studies utilized caught wild individuals and did not account for relatedness in their methylation analyses, which, if not accounted for, may confound the ability to identify environmentally-induced DNA methylation changes [15].

Here we used a controlled experiment to investigate whether hatchery-rearing conditions can influence patterns of DNA methylation in steelhead and directly assessed the stability of these changes over time in fish with known genetic backgrounds. For this experiment, embryos from 22 families of Methow River steelhead were split across two rearing treatments; either traditional hatchery rearing or a simulated stream rearing environment. The families were reared for 8 months before merging the two treatments for an additional 12 months of rearing in a common tank environment. DNA methylation patterns were examined in the liver of male juveniles at the end of the rearing treatment (age-1) as well as in sexually immature males after common hatchery rearing (age-2). Further, we analyzed DNA methylation in the sperm of males that matured at age-2 to assess the potential of environmentally-induced changes to be passed to offspring. With this experimental design, we were able to specifically investigate four questions: (1) Are there immediate changes in DNA methylation after eight months of hatchery rearing? (referred to as *Immediate* hereafter), (2) Are differences in DNA methylation as a result of hatchery rearing persistent after 12 months of rearing in a common environment? (referred to as *Persistent* hereafter), (3) Are there developmental changes in DNA methylation in the same tissue between age-1 and age-2? (referred to as *Developmental* hereafter), (4) What is the potential for intergenerational transmission of DNA methylation changes as a result of hatchery rearing? (referred to as *Intergenerational* hereafter). Addressing this set of questions allowed us, for the first time, to examine the dynamics of hatchery-induced changes in DNA methylation across multiple life-stages and tissues in steelhead; addressing a significant gap in previous studies.

## 2. Materials and Methods

### 2.1. Experimental Design

#### 2.1.1. Overview

**Figure 1:**
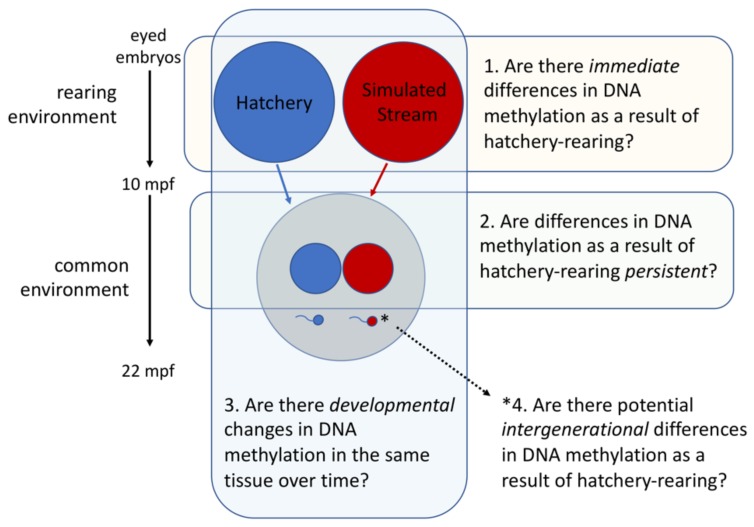
Schematic representation of the experimental design (mpf = months post fertilization).

Eyed-embryos representing 22 *O. mykiss* families were divided equally between a hatchery-rearing treatment and a simulated stream-rearing treatment and reared for eight months at which time a subset of fish were lethally sampled for liver tissue to test for *immediate* differences in DNA methylation (Figure 1, Question 1). After the rearing treatments, the remaining fish were individually tagged by implanting passive integrative transponder (PIT) tags and reared in common hatchery tanks for 12 months, after which a subset of fish was lethally sampled for liver tissue to test for *persistent* differences in DNA methylation (Figure 1, Question 2). Liver samples at both time-points (i.e., *Immediate* v. *Persistent*) were analyzed to test for *developmental* changes in DNA methylation (Figure 1, Question 3). Finally, sperm sampled from males that matured at 20 months was analyzed to test the potential for induced DNA methylation changes to be passed on *Intergenerationally* through the gametes (Figure 1, Question 4). Importantly, the designations of these questions as: *Immediate*, *Persistent*, *Developmental* and *Intergenerational* refer to the question being addressed and not the result we ultimately obtained. Parentage analysis was performed to identify and account for family representation in all sample contrasts. Additional details regarding the experiment are found in the following sections.

#### 2.1.2. Study Population and Crosses

A total of 21 female (19 natural- and 2 hatchery-origin) and 20 male (18 natural- and 2 hatchery-origin) adult steelhead returning to the upper Methow River (near Winthrop, Washington: 48°28’25.1796” N, 120°11’21.6852” W) were used to create 22 families used in this study. Methow River summer-run steelhead are part of an Upper Columbia River evolutionary significant unit (ESU) of West Coast steelhead populations currently listed as threatened under the Endangered Species Act. A majority of the families were single-pair matings, but there was also a single pair of paternal half-sib families and a single pair of maternal half-sibling families. All families had at least one natural-origin parent and a majority of the families (*n* = 18) had two natural-origin parents. Gametes from mature adult steelhead were manually collected and eggs were fertilized using typical hatchery protocols between 15 April and 5 May 2014. Developing embryos were incubated in Heath trays at the Winthrop National Fish Hatchery (WNFH, Winthrop, WA, USA). Incubation temperatures were manipulated to synchronize emergence timing among progeny from different spawning dates to minimize effects of emergence timing on feeding and growth performance after exogenous feeding. Specifically, eggs were held on ambient well water (~7.8 °C) for approximately 2, 7 or 21 days before being slowly (hours) transitioned to chilled (~3.9 °C) water depending on fertilization date (April 15th, April 22nd or May 6th, respectively). See Supplementary Material Dataset S3 for fertilization date by family. Each family was split between simulated stream and hatchery rearing environments for the study, as described below. All fish were reared and sampled according to the University of Washington Institutional Animal Care and Use Committee (Protocol #2313-90).

#### 2.1.3. Stream Rearing Treatment

On 10 June 2014, 40 eyed-embryos per family were collected from their respective Heath trays at the WNFH and placed in a water-saturated paper towel inside of a small plastic container. Each container was placed on a water-saturated cotton towel in an insulated cooler. The containers were covered by another wet cotton towel and the towel was covered with ice cubes. The embryos were transported for approximately 7 h to the Northwest Fisheries Science Center, Manchester Research Station (Manchester, WA, USA) where each family was loaded into a 4-inch diameter PVC isolette. The isolettes were randomly placed into Heath trays. The single stack of Heath trays received approximately 12 L per minute of well water (10.4 °C). The Heath stack was wrapped with black plastic to eliminate most ambient light. The embryos were checked the following day and there were no mortalities.

On 11 June 2014, 40 eggs from each family were divided equally among four containers (i.e., 10 eggs from each family in each of four containers). Groups of 210 eggs from each of the four containers were deposited into four artificially constructed nests in a 40 m-long by 6 m-wide outdoor simulated natural stream channel (described in [31]). The channel was bisected along its length, creating two laterally adjacent sections each consisting of a series of four riffle-pool units separated by 15 cm water falls. Water depths were variable (5–35 cm), current velocities ranged from 0.0 to 0.5 m/s, and gravel size ranged from 1–10 cm diameter. The channel was supplied with well water (80 L/min) that was recirculated at a flow rate of approximately 6800 L/min. A chiller unit was employed during the summer and fall to keep temperatures below 15 °C. Average daily temperatures ranged between 5.6 °C (winter) and 15.7 °C (summer) over the rearing period (see Supplementary Material Figure S1 for average daily temperature of stream). Each nest was buried 20–25 cm and covered with gravel to simulate a nest constructed by a naturally spawning steelhead. Thus, embryos completed development within the interstitial spaces in the gravel, emerged naturally, and began exogenous feeding on natural aquatic insects and other invertebrates, until they were sampled eight months later.

#### 2.1.4. Hatchery Rearing Treatment

On 8 August 2014, juvenile steelhead at the WNFH had reached the “button-up” stage of development; the embryos had utilized all available yolk resources during rearing in typical hatchery stack incubators. At this time, we transferred an additional 20 fry from each of the same families previously stocked in the stream channel at Manchester. The fry were transported to the Manchester Research Station in two insulated coolers, with each cooler containing 10 fry from each female. Each cooler of fry was stocked into a separate circular fiberglass tank (1.8 m diameter × 0.6 m deep) plumbed into a recirculating freshwater system with an annual average rearing temperature of 9.4 °C. Average daily temperatures ranged between 11.6 °C (August) and 7.6 °C (February) over the rearing period (see Supplementary Material Figure S1 for average daily temperature of tanks).

Fry in the hatchery-rearing treatment were fed a size-appropriate pelleted commercial trout diet (BioVita Starter, BioVita Fry, and BioTrout: BioOregon, Longview, Washington, DC, USA). Fry were fed daily to excess for the first three weeks of culture and then fed to satiation five days per week for the remainder of the culture period in order to produce the highest growth rates and highest rates of precocious male maturation possible. Juvenile steelhead in the hatchery-rearing treatment included fish that were also part of another study that required periodic handling for size measurements and tagging. Fish were anesthetized in buffered 0.01% tricaine methanesulfonate (MS222, Argent Chemical Laboratories, Redmond, WA, USA) prior to size measurements in October, November and December 2014, and February and May 2015.

#### 2.1.5. Testing for Immediate Effects of Rearing Environment

In February 2015, a total of 624 fish, 237 stream-reared and 387 hatchery-reared, were weighed (to nearest 0.1 g), measured (fork length (FL) to nearest mm) and implanted with 9 mm PIT tags. Fin clips were taken for DNA isolation to determine genetic sex and family. A subset of the smallest stream-reared individuals (*n* = 117 total (*n* = 53 males)) were too small to PIT tag in February 2015 were subsequently PIT tagged in May 2015 and included in subsequent analyses. A two-tailed t-test was used to detect differences in size between stream- and hatchery-reared individuals.

To address question 1, we examined DNA methylation in liver tissue fish at 10 mpf (referred to hereafter as “age-1” fish). Liver tissue was selected because it is a relatively homogeneous tissue and plays a central role in the endocrine control of somatic growth as well as all metabolic processes controlling storage and mobilization of nutrients. These physiological characteristics are likely to differ between fish reared in hatchery and natural environments because of differences in diet, water chemistry and temperature. Prior to transfer of fish from stream channel to tanks, a subset of 20 fish per treatment were lethally subsampled to collect liver tissue for reduced representation bisulfite sequencing (RRBS) analysis. Fish were anesthetized in 0.01 % MS222 and euthanized by decapitation. Parentage was unknown at the time of liver sampling; fish selected for lethal sampling were size matched as much as possible. Gender was determined by gross morphology of the gonad and genetic sex was determined by analysis for the male sex determining gene (sdY, [32]). A portion of posterior lobe of the liver was dissected and flash frozen in liquid nitrogen until DNA isolation. Only male liver samples from the hatchery- (*n* = 10) and stream-reared (*n* = 10) fish were used for RRBS.

#### 2.1.6. Testing for Persistent Effects of Early Rearing Environment

After subsampling in February 2015, all remaining fish were placed into a common environment (hatchery tanks) for an additional year to test for the persistent effects of hatchery-rearing on DNA methylation in liver of immature males and sperm of males that matured by 20 months of age (referred to hereafter as “age-2” fish). Rearing of fish in the simulated stream environment was limited to eight months because natural productivity was not sufficient to support the biomass of fish as they continued to grow. Hatchery-reared fish were distributed into two tanks (1.8 m diameter, fiberglass) while stream-reared fish distributed into 3 tanks to separate the smallest individuals. The stream-reared fish were then transitioned to a size appropriate pelleted commercial trout diet. Pelleted feed had the same nutritional composition regardless of pellet size. Both the hatchery and stream-reared fish were fed to satiation 5 days a week in order to promote precocious maturation of males in both experimental treatments. In September of 2015, all fish were weighed and measured. A majority of the females (identified by genetic sex test described below) were removed from the study to decrease density and males were distributed back into the tanks. In February of 2016, a subset of immature males was lethally sampled (*n* = 10 each from the hatchery- and stream-rearing treatments) for RRBS analysis in order to assess the persistence of DNA methylation changes in liver tissue resulting from hatchery rearing. A portion of posterior lobe of the liver was dissected and flash frozen in liquid nitrogen until DNA isolation.

#### 2.1.7. Testing for Potential Intergenerational Effects of Early Rearing Environment

To address question 3, males were evaluated for sexual maturation by checking for milt production. All mature males from each treatment were sampled for sperm non-lethally to determine if the early rearing environment affected DNA methylation patterns in a mature gamete. RRBS was performed on the sperm of 30 males from each rearing treatment. At this age, no remaining females were mature.

### 2.2. DNA Based Assays to Identify Genetic Sex and Family

#### 2.2.1. Genetic Sex

Genetic males were identified by the presence of the male sex determining gene, sdY [32], following the procedure described in [33]. Briefly, genomic DNA was PCR amplified using the sdY primers as described by [32]. PCR products were visualized using gel electrophoresis, where the presence of a single band is indicative of a female, duplicate banding is indicative of a male.

#### 2.2.2. Parentage Analysis

Genomic DNA was isolated from finclips of parents and all male offspring using Qiagen DNeasy Kits following the manufacturer’s protocol for isolating DNA from tissues. Isolated DNA was used to genotype 95 single nucleotide polymorphism (SNP) loci, following the methods described by the authors of [34]. The resulting genotypes were used to identify full and half sib family groups by conducting a parentage analysis using the program FRANz [35].

### 2.3. RRBS Library Preparation and Data Analysis

#### 2.3.1. DNA Isolation: Liver

Genomic DNA was isolated from male livers using the DNeasy Blood and Tissue Kit (Qiagen, Valencia, CA, USA) following manufacturer’s instructions. DNA was ethanol precipitated prior to quantification using the Quant-iT PicoGreen dsDNA Assay Kit (Invitrogen, Waltham, MA, USA). A subset of representative DNA samples was analyzed to check DNA quality on the Agilent 2200 TapeStation (Aligent, Santa Clara, CA, USA).

#### 2.3.2. DNA Isolation: Sperm

Milt was expressed by gentle abdominal pressure, collected with capillary action using Natelson tubes, transferred to microfuge tubes and frozen in liquid nitrogen. Genomic DNA was isolated from diluted milt (2 uL milt in 598 uL PBS) using the DNeasy Blood and Tissue Kit (Qiagen) following a user-defined protocol for DNA isolation from sperm. DNA was quantified using the Quant-iT PicoGreen dsDNA Assay Kit (Invitrogen). A subset of representative DNA samples was analyzed for quality on the Agilent 2200 TapeStation (Aligent, Santa Clara, CA, USA).

#### 2.3.3. RRBS Library Preparation

DNA was prepared for RRBS using the gel-free technique as described in [36]. Briefly, 1 ug of genomic DNA was digested with MspI restriction enzyme overnight at 37 °C. Digested DNA was end-repaired, A-tailed and barcoded adapters (TruSeq DNA LT Library Prep Kit (Illumina, San Diego, CA, USA)) were ligated prior to pooling (*n* = 7–19 samples per pool). Two rounds of bisulfite conversion were performed using the EpiTect Bisulfite Kit (Qiagen), followed by PCR amplification (TruSeq DNA LT Library Prep Kit (Illumina)) and final clean-up using Agencourt AMPure XP beads (Beckman Coulter). Pooled libraries were sequenced for 100 cycles (single-end reads) in 2–4 lanes (depending on the number of individuals in the pool) on an Illumina HiSeq. The *Immediate* and *Intergenerational* samples were sequenced on a HiSeq 2500 (*Immediate* libraries were sequenced at Omega Bioservices (Norcross, GA, USA)) and *Intergenerational* libraries were sequenced both Omega Bioservices and the University of Oregon, Genomics and Cell Characterization Core Facility (Eugene, OR, USA). *Persistent* libraries were sequenced on a HiSeq 4000 at the University of Oregon, Genomics and Cell Characterization Core Facility. Sequencing data are available in the NCBI SRA database under the BioProject PRJNA525072.

#### 2.3.4. Sequence Trimming and Mapping

Sequencing reads were quality and adapter trimmed using *TrimGalore* (http://www.bioinformatics.babraham.ac.uk/projects/trim_galore) a wrapper for the publicly available trimming tool *cutadapt* [37] and FastQC [38]. TrimGalore (v0.4.4) was run with default parameters and the additional RRBS specific option *--rrbs*. Trimmed reads were aligned to the *O. mykiss* reference genome scaffolds (GCF_002163495.1 [39]) with the bisulfite mapping tool Bismark v0.18.0 [40]. Bismark used Bowtie2 for mapping with the function for minimum score alignment set to allow approximately 3 mismatches per 100 bp read (option: *--score_min L,0,*–*0.2*). Count data for methylated and unmethylated reads were extracted using the *Bismark methylation extractor* script for downstream analysis. In order to screen for batch effects between sequencing platforms and facilities, bisulfite treatment efficiency was estimated using non-CG methylation and a two-tailed *t*-test was performed. Furthermore, all libraries were evaluated with FastQC and reports visualized using multiQC [41] to identify potential batch effects.

#### 2.3.5. Identifying Differences in DNA Methylation

Differential methylation analyses were performed for each of the four questions/sample contrasts: *Immediate* (*n* = 10 each per rearing group), *Persistent* (*n* = 10 each per rearing group), *Developmental* (*n* = 20 each per time-point), *Intergenerational* (*n* = 30 each per rearing group). Filtering steps and differential methylation analyses, described in detail below, were performed for each contrast separately in order to retain the highest number of CG sites for differential methylation analyses.

For each contrast, CG sites that were covered in at least half of the samples per rearing group were considered for analysis. In addition, CG sites with median coverage ≤10, CG sites that had ≥90% or ≤10% average DNA methylation across all samples or were in the lowest 5% of variance in methylation among samples were filtered out prior to modeling differential methylation. Due to the inability of bisulfite sequencing technologies to discern a true C/T SNP from a methylation change, a final filtering step was performed to remove sites with a putative CG SNP. BS-SNPer [42] was used with default parameters to identify SNPs in the RRBS data. If a CG SNP was identified in any of the individuals used in this study, the site was removed from further analysis. Differential methylation analysis was performed on all CG sites passing filtering using the beta-binomial mixed model “MACAU” [43], which includes genetic relatedness as a random effect. A genetic relatedness matrix was generated for all individuals using the R package *StAMMP* [44] based on the SNP data used for parentage analysis. MACAU was performed using rearing-environment (hatchery or stream) as the predictor variable for the *Immediate*, *Persistent* and *Intergenerational* sample contrasts. For the *Developmental* contrast, time-point (age-1 or age-2) was used as the predictor variable and rearing-environment was used as a model covariate to account for differences that may be specific to a given rearing environment. Multiple hypothesis testing was performed on *P*-values extracted from the MACAU output for each CG site using the false discovery rate (FDR) approach used in the R package *qvalue* [45]. We considered a CG site to be differentially methylated (i.e., differentially methylated cytosine (DMC) if it passed a 10% FDR threshold, consistent with [46]. We also compared DMCs between the *Immediate* and *Persistent* sample contrast. Because CG sites were filtered separately for each contrast, we evaluated the amount of overlap between CGs analyzed between these sample contrasts. The genomic location of all CG sites analyzed as well as DMCs for the *Immediate* and *Persistent* time-points were compared for overlap using the R package *dplyr* [47].

We also identified differentially methylated regions (DMRs)—spatially co-located stretches of differentially methylated CGs, which are more likely to regulate gene expression than differentially methylated sites occurring in isolation [48]. Meeting one of the following two criteria was required for a region to be identified as a DMR: (1) Two or more CG within a 2 kb region centered on a DMC with a *P*-value of ≤0.001, or, (2) three or more CG sites with a *P*-value < 0.001 within a 2 kb region.

All DMCs and DMRs were annotated to genes using predicted gene models for the *O. mykiss* reference genome (NCBI assembly accession: GCF_002163495.1). DMCs and DMRs overlapping genes and their putative cis regulatory domains (defined here as within 10 kb of the transcription start site or transcription end site) were identified using the software BEDTools [49]. This annotation window was selected to include both proximal promoters as well as distal regulatory regions that may be epigenetically regulated (e.g., [50,51]). Genes were aligned to the UniProtKB/Swiss-Prot database (http://uniprot.org) in order to determine homology to known protein sequences. Alignments were made using the BLAST algorithm [52] (blastx with 1 × 10^−10^ e-value cutoff). Pathway and network analysis were conducted on genes associated with DMCs using Qiagen’s Ingenuity Pathway Analysis (IPA). To analyze in IPA, genes were assigned orthologs using the ENSEMBL zebrafish (GRCz10 release 84), ENSEMBL human (GRCh38 release 84), and Swiss-Prot databases using BLAST (v2.6.0+). Orthologs were then checked with IPA’s internal database to find a match. If multiple databases had a match, the order of selection was zebrafish, human then Swiss-Prot. A gene was only assigned an ortholog if IPA found a match with the ortholog’s accession number and the BLAST *e*-value was ≤ 1 × 10^−5^. All genes within 10 kb of an analyzed CG site were used as the background for enrichment analysis. Significantly altered pathways were defined as those with a Fisher’s Exact Test (*P*-value < 0.05) with a minimum number of hits per pathway cutoff of 3.

## 3. Results

**Figure 2:**
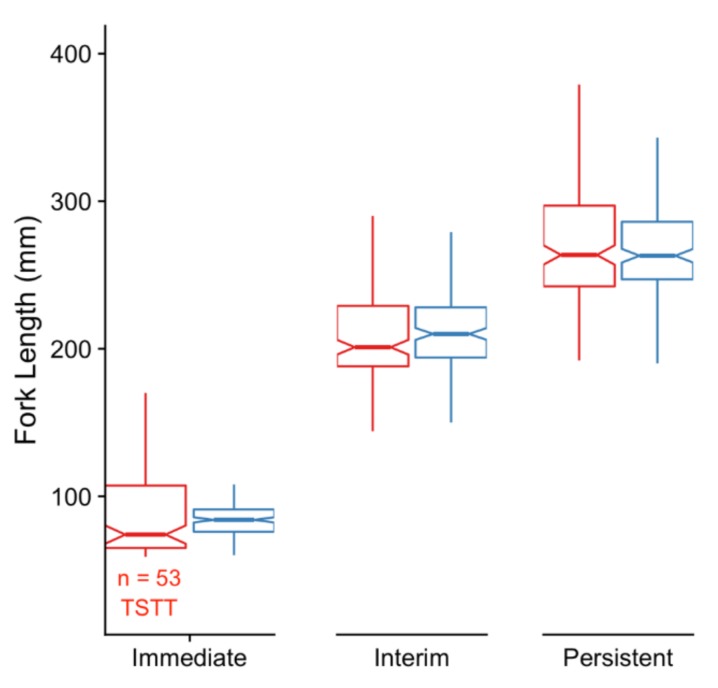
Fork length measurements of male fish that experienced early-rearing in either a stream (red) or hatchery (blue) environment at the *Immediate* (Feb 2015) and *Persistent* (Feb 2016) sampling time-points as well as an Interim time-point in September 2015. Fifty-three stream-reared males were not measured at the *Immediate* time-point as they were too small to tag (TSTT). The TSTT fish were subsequently tagged and measurements for these fish are included for the Interim and *Persistent* time-points.

At the *Immediate* sampling point, there were no significant differences in body weight or FL between males and females (data not shown), but stream-reared males generally tended to be shorter than hatchery-reared males and also showed more variation (Figure 2). At the *Persistent* time point, there were no significant differences in body weight or FL of fish from the hatchery or stream-reared treatments (Figure 2).

DNA methylation patterns were analyzed using RRBS to characterize the dynamics of hatchery-induced DNA methylation changes. An average of 40 million, 100 bp single-end reads per individual were obtained for each RRBS library. Only one sample, a hatchery-reared fish from the *Persistent* time-point, had fewer than 10 million reads and was dropped from further analysis. Estimated bisulfite conversion efficiencies and library profiles were similar between libraries sequenced on different platforms/facilities. Total mapping efficiency of the RRBS reads to the reference genome was 83%, but only 53% mapped uniquely and were used for subsequent analysis. Multimapping reads are not uncommon in bisulfite sequencing given the reduced complexity of the genomic DNA following bisulfite treatment, however, it is also important to acknowledge other factors that may be contributing to non-unique mapping in this study. Specifically for steelhead and other salmonids that underwent whole genome duplication, regions of the genome that are experiencing late rediploidization may be underrepresented because short sequencing reads may align equally well to the duplicated regions [53,54]. Supplementary Material Dataset S1 for detailed sequencing and mapping information for individual samples.

In order to retain as many CG sites for analysis as possible, filtering criteria were assessed on each of the 4 sample contrasts (i.e., *Immediate*, *Persistent*, *Developmental* and *Intergenerational*) separately. Table 1 provides the results of filtering steps performed prior to differential methylation analysis for each sample set. It is important to note that the *Intergenerational* sample set used DNA from sperm where a large proportion of the CG sites had average methylation >90% across all samples. These CG sites were filtered out prior to differential methylation analysis in MACAU, and as a result, only a quarter of the number of CG sites were interrogated for this sample set. Bisulfite sequencing is the gold standard of DNA methylation analysis, but it cannot differentiate between C/T SNPs. Here, approximately 20,000 CG sites were identified as possible C/T SNPs across the sample sets. These CG sites were removed from further analysis.

### 3.1. Immediate Effects: Differences in DNA Methylation in the Liver of Hatchery vs. Stream-Reared Males (Age-1 Fish)

**Figure 3:**
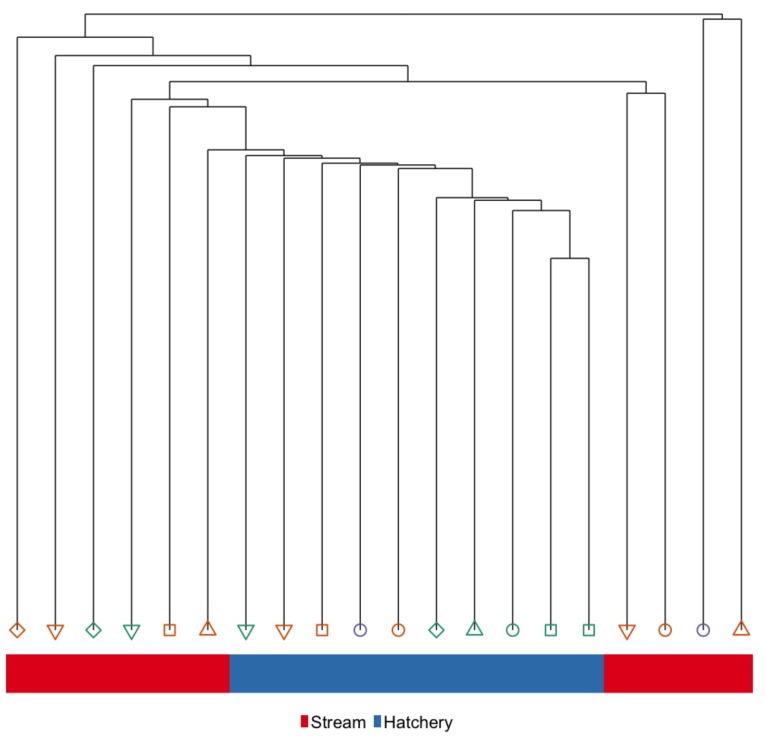
Hierarchical clustering of global hepatic DNA methylation patterns in the *Immediate* sample set. Color of the bar represents rearing-treatment and color/shape represents family.

Body size (weight and FL) of fish used in the *Immediate* sample contrast (*n* = 10 per treatment) set were not significantly different between rearing groups. Eleven families were represented within this sample set; 6 of the families were represented in both hatchery and stream treatments. See Supplementary Material Dataset S2 for body size and family assignments. Hierarchical clustering of global DNA methylation patterns in liver tissue distinguishes hatchery and stream-reared fish, and while all hatchery fish cluster together, the stream-reared fish form multiple sub-groups, some of which cluster more closely with the hatchery-reared fish (Figure 3). Furthermore, with only one exception, siblings did not cluster closely together in the liver at the *Immediate* time-point.

**Figure 4:**
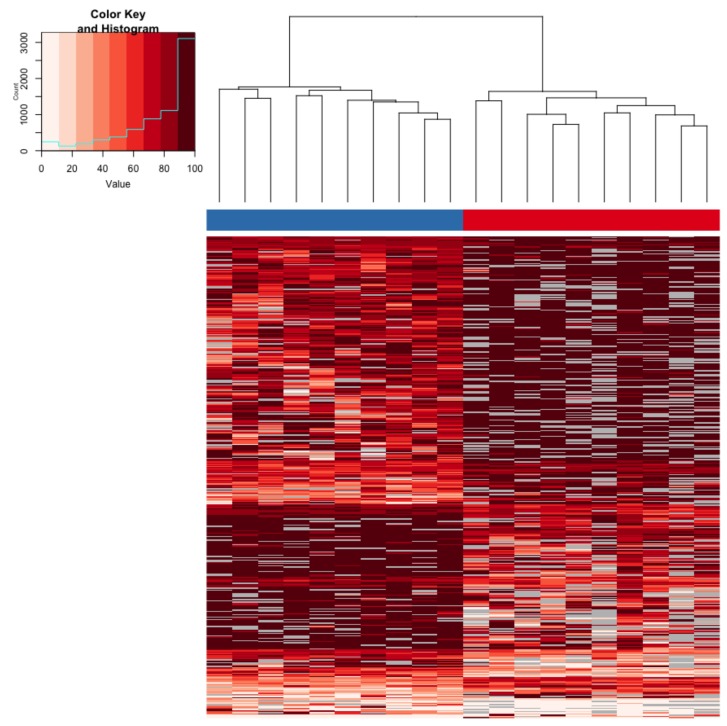
Hierarchical clustering of hepatic differentially methylated cytosines (DMCs) from the *Immediate* time-point. The rearing-group is identified by color (hatchery = blue, stream = red) at the top of the column. Each row represents a DMC. The heatmap depicts percent methylation for each DMC for each individual with the darkest red indicating 100% methylation and the lightest indicating 0% methylation. The regions that did not meet the coverage cutoff for a particular individual are represented by gray boxes.

Analysis of differential liver methylation identified 413 differentially methylated cytosines (DMCs) between hatchery- and stream-reared fish. The average absolute difference in methylation for DMCs was 25.7% (range: 8.3–65.5%). More than half of the DMC (*n* = 251) were hypo-methylated in hatchery-reared fish compared to stream-reared fish. Individuals clustered according to rearing-environment based on the 413 DMC (Figure 4). Almost 75% of the of the DMCs (295) were associated with genes (Supplementary Dataset S3). Gene ontology enrichment analysis identified 31 enriched biological pathways in genes associated with these DMC (Supplementary Material Table S1). We identified 10 spatially co-located stretches of DMCs referred to as differentially methylated regions (DMRs). The average difference in methylation in DMRs was 23% (range: 15–40%) with an average number of CGs making up a DMR of 3 (range: 3–4). Eight of the DMRs were located in close proximity to genes (Supplementary Dataset S3).

### 3.2. Persistent Effects: DNA Methylation Differences in the Liver of Hatchery vs. Stream Reared Males After One Year in a Common Environment (Age-2 Fish)

**Figure 5:**
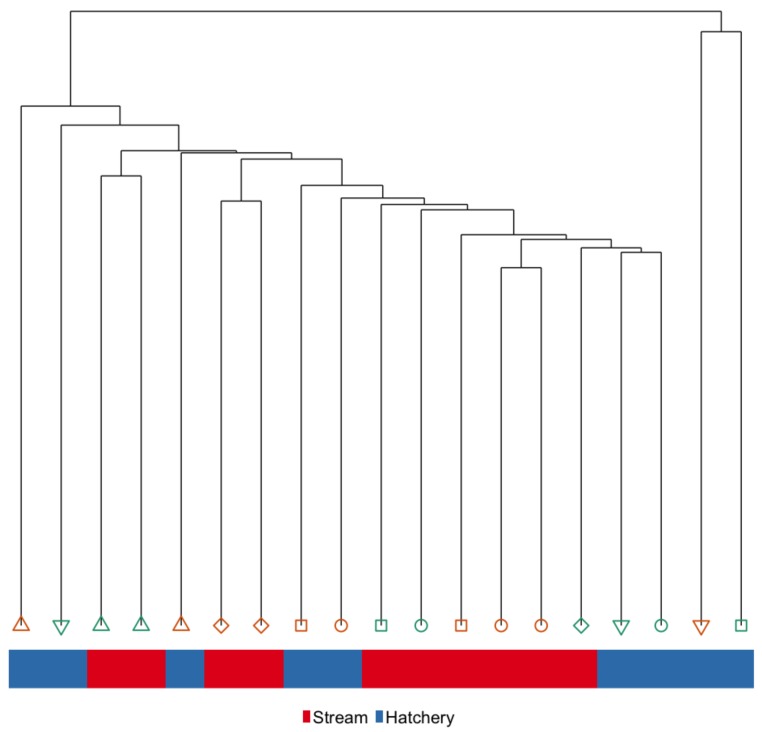
Hierarchical clustering of global DNA methylation patterns in the *Persistent* sample set. Color of the bar represents rearing-treatment and color/shape represents family.

There were no differences in body weight or FL between hatchery- (*n* = 9) and stream-reared (*n* = 10) male fish at the *Persistent* time-point. Ten families were represented within this sample set; 4 of the families were represented in both the hatchery and stream rearing groups. See Supplementary Material Dataset S2 for size and family assignments. Hierarchical clustering of global DNA methylation patterns in liver tissue at the *Persistent* time-point does not distinguish hatchery and stream-reared fish, in contrast to the sample set from the *Immediate* time-point (Figure 5).

Next, we performed an analysis of differential methylation within this sample set in order to identify DMCs independent of the previous, *Immediate*, time-point. Differential methylation analysis identified 396 DMCs in the *Persistent* sample set (Supplementary Dataset S3). The average absolute difference in methylation for the DMCs was 27.7% (range: 11.0–77.8%). Less than half of the DMCs (140) were hypo-methylated in fish that had experienced hatchery-rearing in the first year compared to stream-reared fish. A majority of the of the DMCs (268) were associated with genes (Supplementary Dataset S3). Gene ontology enrichment analysis identified 14 enriched biological pathways in genes associated with these DMC (Supplementary Material Table S2). Six DMRs were identified in the sample contrast (Supplementary Dataset S3).

**Figure 6:**
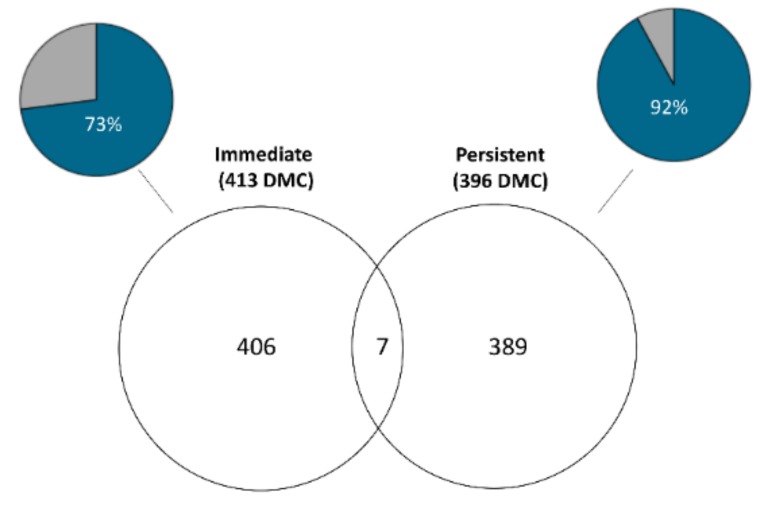
Venn diagram of hepatic DMCs that overlap between *Immediate* and *Persistent* time-points. Pie charts indicate the proportion of the DMCs that met coverage criteria (blue) in order to be considered for DMC analysis in both time-points, the proportion of DMCs in grey were not tested in both time-points.

Then we compared the DMCs identified in the *Persistent* sample set to those of the *Immediate* sample contrast to identify overlaps. Although 73 and 92% of DMCs met the minimum coverage criteria for the *Immediate* and *Persistent* sample contrasts, respectively (Figure 6), only a single overlapping DMC was identified. An additional 6 DMCs were within 10 kb of each other between the *Persistent* and *Immediate* sample sets (Figure 6). The direction of methylation change was consistent between the *Immediate* and *Persistent* sample sets in only one of the seven proximal DMCs (Supplementary Material Table S3).

Given the limited evidence to suggest a persistent signal of hatchery rearing in the liver after a year of rearing in a common environment, we examined developmental changes in hepatic DNA methylation between the *Immediate* (age-1) and *Persistent* (age-2) sample sets by analyzing DMCs using treatment as a covariate.

### 3.3. Developmental Effects: Differences in DNA Methylation in the Liver of Age-1 vs. Age-2 Males

**Figure 7:**
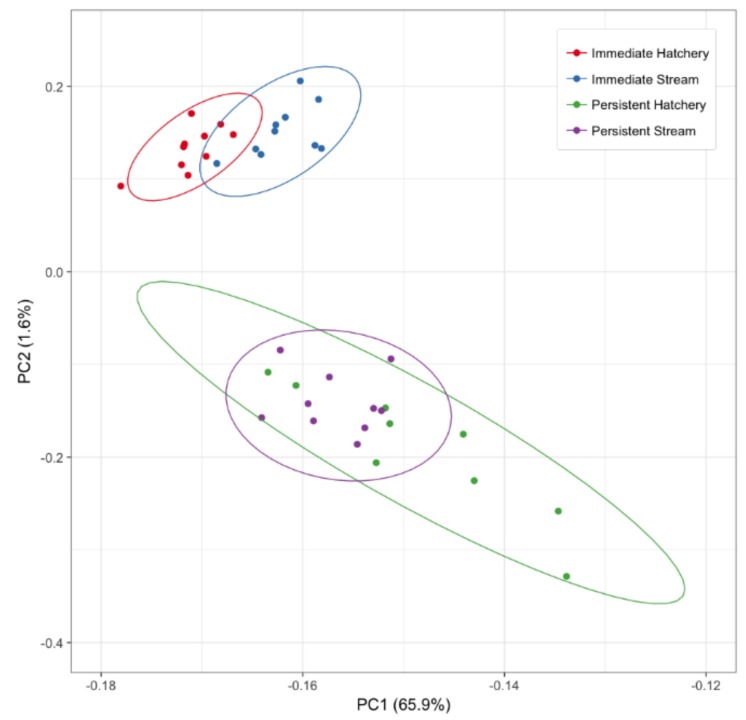
Principal components 1 and 2 of PCA describing variation in hepatic DNA methylation between rearing-group (hatchery or stream) and time-point (*Immediate* (age-1) or *Persistent* (age-2)).

We performed a PCA on the DNA methylation data from the *Immediate* (age-1) and *Persistent* (age-2) sample sets. Figure 7 shows the first and second principal components resulting from the PCA which indicates the variation and degree of differentiation in DNA methylation among groups. The first component, accounting for 66% of the variance, identified the variation between the stream and hatchery-reared fish at the *Immediate* time-point, but also indicates separation between the *Immediate* and *Persistent* time-points. The fish that experienced early rearing in the stream cannot be distinguished from those that experienced early rearing in the hatchery at the *Persistent* time-point. The second principal component, accounting for 1.6% of the variation further separates the *Immediate* and *Persistent* time-point. DNA methylation patterns displayed global significance between the rearing groups and time-points (ANOSIM, *R* = 0.128, *P*-value = 0.001)

We also performed a differential methylation analysis between the *Immediate* (age-1) and *Persistent* (age-2) samples using rearing environment as a model covariate and identified 25,513 DMCs (Supplementary Table S1). The average absolute difference in methylation for DMCs was 18.2% (range: 1.6–77.8%) and a vast majority of the DMCs (23,458) were hypo-methylated in the *Persistent* sample set, where fish are 1 year older than the *Immediate* sample set. Almost 75% of the of the DMCs (18,255) are associated with genes (Supplementary Dataset S3). Gene ontology enrichment analysis identified 243 enriched biological pathways in genes associated with these DMCs (Table 2 and Supplementary Material Table S4).

There were 403 DMRs identified. Only 63 DMRs were hypomethylated in *Persistent* (age-2 relative to age-1) samples, but these DMRs were the most extreme in terms of having some of the largest absolute differences in methylation. For example, Table 3 highlights the 17 DMRs with absolute methylation difference greater than 40%. All of them are hypomethylated in the age-2 time-point and a majority of them contain 5 or more CGs that are within 5 kb of genes. All *Developmental* DMRs are reported in Supplementary Table S4.

### 3.4. Intergenerational Effects: Differences in DNA Methylation in the Sperm of Hatchery vs. Stream-Reared Reared Fish After One Year in a Common Hatchery Environment

We performed DNA methylation analysis on sperm of fish that experienced early rearing in either the hatchery or stream environment, but sexually matured in a common environment, to test for the potential intergenerational effects of rearing environment on DNA methylation patterns. A similar proportion of males from the hatchery and stream rearing group, 18% and 20% respectively, matured at age-2. RRBS was performed on 30 males from each group. Nineteen families were represented within this sample set; eight families were represented in both hatchery and stream treatments. (see Supplementary Material Figure S3 1 for proportion of males maturing at age-2 by family). The body size of fish within this sample set were not significantly different between treatments. See Supplementary Material Dataset S2 for body size and family assignments.

**Figure 8:**
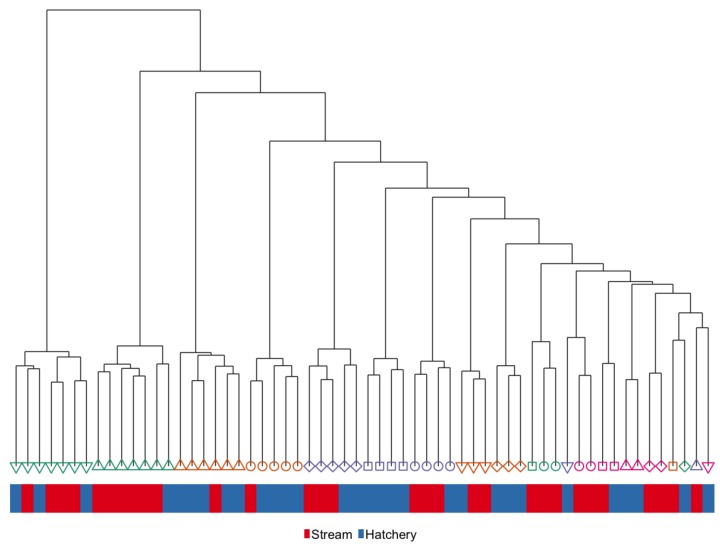
Hierarchical clustering of global DNA methylation patterns in the *Intergenerational* sample set. Color of the bar represents rearing treatment and color/shape represents family.

DNA methylation levels were constitutively high across all sperm samples, resulting in far fewer CG sites being analyzed than for liver samples (Table 1). We performed hierarchical clustering of global DNA methylation patterns in sperm and found that individuals did not cluster according to rearing group, but instead, clustered very tightly by family, with siblings exhibiting more similar methylation patterns than unrelated fish (Figure 8).

Differential methylation analysis was performed on these samples and 19 DMCs were found between hatchery and stream-reared fish. The average absolute difference in methylation was 20.7% (range: 7.3–39.5%). Approximately half of the DMCs were hypomethylated in fish that experienced early-rearing in the hatchery environment. Fourteen of the DMCs were associated with genes (Supplementary Table S4). The DMCs do not cluster according to treatment, again highlighting the strong family effect in this sample set (Supplementary Material Figure S4). Eight DMRs were identified in sperm. The average absolute difference in methylation in DMRs was 26% and 5 of 8 were hypomethylated in the hatchery-reared fish. Seven of the DMRs were located in close proximity of genes (Supplementary Dataset S3). There were no significantly enriched biological pathways associated with these DMCs.

We evaluated the 19 DMC in sperm to determine if any of these CG sites were also differentially methylated in the liver of the *Immediate* and *Persistent* datasets. There were no overlaps with DMCs identified in the *Immediate* sample set, but one sperm DMC, that made up a larger DMR, was within 60 bp of a DMC identified in the *Persistent* sample set. These proximal DMCs, taken from different tissues at the same time-point, were both hypomethylated in fish that had an early hatchery-rearing environment. These CG sites are located on Chromosome 24, approximately 3 kb upstream of a *Heat shock cognate 70 kDa protein* gene.

## 4. Discussion

To minimize the loss of fitness phenotypes that have been observed in hatchery-reared steelhead, a better understanding of how the hatchery environment is affecting subsequent life-history and fitness traits as well as the underlying mechanisms is needed. Here we used a lab-scale study to investigate the dynamics of hatchery-induced DNA methylation changes in fish with known genetic background. The aim of our study was to investigate the potential of DNA methylation to ‘store’ information about the hatchery-environment in the form of an epigenetic memory. This is the first study to examine the effects of captive rearing across life stages and tissues in fish with known relatedness. Results suggest that initially DNA methylation changes in response to hatchery-rearing are substantial, but that developmental and environmental signals continue to shape the methylome, in a sense “overwriting” the original signal of the hatchery environment. Nevertheless, there are differences in DNA methylation patterns between hatchery- versus stream-reared fish after a year of common rearing. While these differences are not “persistent” *per se*, they may be reflecting how physiological responses at this later time-point may have been shaped by the early environment indirectly. Small differences observed in the sperm methylome may suggest limited potential for intergenerational changes, but care should be taken in this interpretation, as only a fraction of the genome is analyzed in RRBS and the high degree of methylation in this cell-type makes small changes difficult to detect. The effect of family observed in the DNA methylation patterns in sperm were striking and highlight the importance of accounting for relatedness in studies of DNA methylation in natural populations.

### 4.1. Immediate

We identified substantial DNA methylation differences in the livers of juvenile male steelhead that had experienced a majority of their early rearing in a hatchery compared to stream environment. Global analyses of the liver methylome showed separation between the hatchery- and stream-reared fish when sampled after 8 months of rearing in the two environments (Figure 3 and Figure 7). Our results are in accordance with a recent study in juvenile coho salmon that showed robust differences in DNA methylation in muscle of hatchery- versus natural-origin juveniles from two independent river systems [26]. In addition to global methylation analysis, we also identified 413 DMCs between hatchery- and stream-reared fish at this time-point, a majority of which were associated with genes. Interestingly, several genes associated with DMCs in the liver in the current study were also found to be differentially methylated in muscle of hatchery-origin juvenile coho salmon [26]. For example, two genes, *TATA-binding protein-associated factor 172* and *DnaJ homolog subfamily C member 17*, were both found to be hypermethylated in hatchery-reared juveniles, perhaps suggesting methylation patterns associated with these genes may be more general responses to hatchery rearing in juveniles, while methylation changes in other regions may be more hatchery, tissue or species-specific. TATA-binding protein-associated factors are associated with activating RNA polymerase III as part of a complex with TATA-binding proteins [55], while DnaJ proteins (also referred to as heat-shock protein 40), act as co-chaperones to the 70 kDa heat-shock proteins (Hsp70), which play a role in modulating protein folding, assembly, disassembly and translocation [56]. A gene within the same family, *DnaJ homolog subfamily C member 21*, was shown to be differentially regulated in the offspring of hatchery versus wild steelhead that had been reared in a common environment [4] perhaps indicating a relationship between methylation and expression of this gene in response to hatchery-rearing. Enrichment analysis using IPA identified several biological pathways that were significantly enriched (Supplementary Table S4), including the epidermal growth factor (EGF) signaling pathway, which is involved in cell proliferation and survival in the liver [57]. Differentially methylated genes in this pathway include serine/threonine kinases (*AKT serine/threonine kinase* 1 and *protein kinase C alpha*) and genes capable of binding EGF (*growth factor receptor-bound protein 2*, *inositol 1,4,5-trisphosphate receptor type 3)*. Although additional research would be required to understand the direct relationship between DNA methylation changes and gene expression changes in the current study, these results highlight biological pathways, such as those related to liver development and injury, may be impacted by hatchery rearing.

In many salmon and steelhead hatcheries, environmental conditions in the hatchery are manipulated in order to accelerate growth. In particular, temperature and diet/feeding rate during early life-history can be very different than environmental conditions experienced by their wild conspecifics [12,58]. Therefore, perhaps it is unsurprising that substantial differences in DNA methylation are observed in metabolically active tissues like the liver or muscle in hatchery-reared juvenile salmonids at a time-point consistent with when they would be released into the wild. Because DNA methylation can influence the expression of genes [59], and temperature and diet in particular have been repeatedly shown to influence DNA methylations patterns (e.g., [21,60,61]), differences at this time-point may be reflecting the differing physiology required to perform optimally in the hatchery versus natural environment. Although it is possible that hatchery-reared fish may utilize the natural environment differently than a wild fish due to behavioral or other physiological phenotypes [62], once steelhead are released from the hatchery at 1 year of age, they will essentially share the environment with wild counterparts for a majority of their lives (typically 1–4 years [63]) before they reproduce. Therefore, a critical question to address, with respect to the role of DNA methylation in mediating loss of fitness phenotypes associated with hatchery rearing, is how persistent or plastic are these DNA methylation differences over developmental time in a shared environment?

### 4.2. Persistent

Temporal stability of environmentally-induced DNA methylation programming is an often-overlooked area of study. In this study, we tested whether hatchery-induced DNA methylation differences were reversible or persistent by analyzing methylation in the liver of hatchery- and stream-reared males after a year in a common environment. Only sexually immature males were used to avoid confounding effects of maturation. Hierarchical clustering of global DNA methylation levels in the liver of age-2 males (termed the *Persistent* sample contrast) no longer distinguished hatchery versus stream-reared individuals as they did in the age-1 (i.e., *Immediate*) males. This is consistent with a previous study that also failed to identify global differences in DNA methylation between adult hatchery and natural-origin steelhead [64]. However, the approaches used by Blouin et al. [64] did not have the resolution to detect the fine-scale differences in methylation. In the current study, using RRBS, we identified a similar number of DMCs at both the *Persistent* and *Immediate* time-points. The DMCs we identified in livers after a year of common rearing were not “persistent” *per se* as we found a vast majority of the DMCs identified were unique compared to those identified in the *Immediate* sample contrast. It appears that although the vast majority DNA methylation changes observed immediately post treatment were reversible, a signal of early-rearing environment was still present in the DNA methylation patterns of the liver in age-2 fish, albeit at new, or not previously identified, genomic loci. This suggests that the early rearing environment affected the response to the subsequent rearing environment, but a greater understating of molecular mechanisms underlying how the early-environment continues to influence the dynamic DNA methylation landscape over developmental time is still needed.

Despite the fact that over 70% of the DMC identified in livers of the males in the *Immediate* sample contrast were analyzed in the *Persistent* sample contrast, very few DMC overlapped between the time-points (Figure 6). We acknowledge that a whole genome bisulfite sequencing approach could have detected more common DMCs in regions of the genome not covered by RRBS. Nonetheless, if we assume the regions covered are representative of the whole genome, our data indicate that a majority of the DMCs were unique to each time-point. In our analysis, only 7 DMCs were overlapping or within close proximity between the age-1 (*Immediate*) and age-2 (*Persistent*), and of those, only a single locus, associated with the gene *Dystrophin*, showed a consistent direction of methylation change (hypomethylated in hatchery- versus stream-reared fish) at both time-points. *Dystrophin* has been shown to be differentially expressed in liver of trout fed an animal- versus a plant-based diet [65]. In mammals, it has been shown repeatedly that diet quantity, quality and composition during development can have lasting effects on adult phenotypes which are also associated with long-term changes in DNA methylation [66]. One could speculate that, similar to mammals, the differences in methylation observed in the liver in the *Persistent* sample contrast could be evidence that early differences in diet (artificial pellets v. natural invertebrates) between hatchery and stream-reared fish are being reflected in the liver even after a year of common rearing.

This experiment was designed to mimic the early-life conditions experienced by hatchery- and wild-reared steelhead; however, due to capacity restraints on the simulated stream, once the rearing treatment was complete, just 2 months prior to the time when hatchery steelhead are released into the wild, fish from both treatments were placed into tanks for a year of common rearing. It is likely that DNA methylation patterns observed in the liver after one year in the tank environment on artificial feed reflect transcriptional and physiological responses to diet, temperature, water chemistry, and density of the fish in the tank among other factors. While the methylation profiles in the age-2 fish are likely not reflective of DNA methylation patterns of similarly aged steelhead in the wild, the biological function of differentially methylated genes at this time-point is applicable to investigations into nutritional and other types of developmental programming in aquaculture. For example, controlled stress exposure in early-life may promote growth in Atlantic salmon [25,67], and early exposure to a plant-based diet improved the acceptance and utilization of the same diet at a later life stage in rainbow trout [22]. A study examining DNA methylation in the blood of wild-feeding (resource limited) and lodge-feeding (resource abundant) baboons, showed that that DNA methylation patterns of individuals that switched resource base between wild- and lodge- feeding at any life-stage more closely matched DNA methylation patterns in exclusively wild-feeding individuals, suggesting that exposure to a challenging, resource-limited environment leaves a stronger signature in the methylation memory than favorable environments [46]. In the future, it would be interesting to carry out the current study in steelhead utilizing a simulated “natural” common environment in order to determine if more or fewer of the hatchery-induced changes would be retained when the novel environment was resource-limited.

In general, the data from the present study support the notion of plasticity over persistence in DNA methylation differences induced by hatchery rearing, however, this does not tell the whole story as new DMCs were identified at this time-point. The lack of differentiation between the hatchery and stream-reared fish at the *Persistent* time-point may be reflecting greater overall variation among individuals in DNA methylation. Indeed, there is clearly larger variation in liver DNA methylation patterns among the *Persistent* (age-2) compared to the *Immediate* (age-1) individuals according to principal component analysis (Figure 7). This may be due to physiological/developmental changes in the liver that are initiated in the spring, and affected by rearing history and age (see discussion below). In February 2015, fish could have been at various stages of initiating smoltification based on their growth history and age. Although the fish could not be globally differentiated based on methylation, we still identified DMCs and DMRs at this time-point indicating latent effects of hatchery rearing on the methylome that are present at this life-history stage. Hatchery-rearing conditions have been reported to influence smoltification rates [58,68] and this could be reflected in the DNA methylation patterns in a number of tissues at this stage. We looked into this further by comparing the liver DNA methylation patterns between the age-1 (i.e., *Immediate*) and age-2 (i.e., *Persistent*) males directly.

### 4.3. Developmental

DNA methylation plays an important regulatory role during development and methylation states are known to change with developmental stage [69] as well as age [70,71]. Anadromous salmon and steelhead undergo developmental (morphological, physiological and behavioral) changes prior to and during their seaward migration via a process called smoltification [72,73,74]. Anadromy in steelhead is a heritable trait [75] and associated with specific DNA sequence variation in the genome [76], brain gene expression profiles [77], and DNA methylation patterns in fin tissue [78]. However, smoltification is a threshold trait where rearing practices that alter growth can affect the age of smoltification [68,79,80]. In steelhead, smoltification occurs in the spring [81], primarily at age-2 (or older) in natural-origin fish [12]. Indeed, the most extreme differences in DNA methylation in our comparison between livers of age-2 (i.e., *Persistent*) and age-1 (i.e., *Immediate*) immature male steelhead, could be associated with smoltification. Global methylation analysis using PCA clearly separates age-1 and age-2 individuals (Figure 7), and differential methylation analysis using rearing-treatment as a covariate identified tens of thousands of DMCs. Overwhelmingly, DMCs were found to be hypomethylated in the livers of age-2 compared to age-1 males. In addition, the most extreme DMRs, in terms of absolute difference in methylation (≥40%), were all hypomethylated in the age-2 livers (Table 2). Interpreting the functional outcome of hypomethylation of the DMRs is speculative without corresponding gene expression data. Typically, hypomethylation is associated with increased gene expression [82] but this relationship is also dependent on the genomic context [83]. Aging has also been associated with general trends toward hypomethylation [84], but not at all aging-related loci become hypomethylated with age [85] and patterns of methylation due to aging are relatively unstudied in non-mammalian vertebrates [86]. Additionally, changes in DNA methylation in the liver may also reflect changes in the proportion of cell types in the liver between these two time-points; however, this may play a smaller role in liver which is made up primarily of hepatocytes [87].

Enrichment analysis of genes associated with DMCs from the *Developmental* sample contrast highlight possible biological function of these differences. Our analysis with IPA identified 98 canonical pathways with a *p* value <0.001 and 60–87 % of the genes in each pathway are in proximity to DMCs between age-1 and age-2 fish. Many of these pathways have been associated with physiological changes during smoltification, which include elevated metabolism, energy production, body elongation, fat loss, and changes in hemoglobin and ion regulation [88]. These changes are promoted by thyroid hormones (THs), growth hormone (Gh), insulin-like growth factors (Igfs) and cortisol [89]. The liver develops increased capacity for glycolysis, lipolysis and decreased lipid biosynthesis [90], increased TH [91] and Gh [92] signaling, and increased Igf production [93]. While our study was not intended to characterize changes in liver methylation during smoltification, the observed differences in methylation in livers of age-1 and age-2 fish could be related at least in part to differences in smolt status since pathways involved in lipid metabolism, cell development, Igf1 signaling, glucose metabolism, TH signaling, etc. were identified (Supplementary Material Table S4). Among some of the top pathways identified as being enriched was PPARαRXRα Activation. PPARs form heterodimers with retinoid X receptors (RXRs) that bind to specific PPAR response elements in the regulatory regions of target genes and act as transcriptional regulators. PPARs, which are activated by fatty acids and their derivatives, regulate lipid and glucose metabolism and affect cellular proliferation, differentiation and apoptosis [94,95]. Although we did not measure expression of hepatic genes in the current study, the observed differences in methylation are consistent with some of the transcriptomic differences in the livers of coho salmon (*O. kisutch*) parr and smolts [96].

While the comparison of DNA methylation between different life stages in steelhead does not directly address questions related to the role of epigenetics in mediating hatchery-induced phenotypes, it does highlight an important aspect of study design and biological interpretation that needs to be considered. This comparison does highlight the temporal plasticity of DNA methylation regardless of environment. DNA methylation appears to strike a balance between stability and plasticity; therefore, inferring the stability of environmentally-induced DNA methylation changes based on a single time-point or life stage may be misleading.

### 4.4. Intergenerational

The period of primordial germ cell differentiation into oogonia or spermatogonia has been shown in mammals to be a period when environmental factors may influence DNA methylation patterns of the germ line, and could potentially be passed to subsequent generations [97]. We hypothesized that early rearing environment in the hatchery may affect epigenetic programming of the germ line because the period of sex differentiation in salmonid fishes occurs at the alevin stage [98]. In the present study, fish were in their respective treatments during the critical period of sex differentiation (approximately two months post fertilization). Compared to the liver, only a small number of differences in DNA methylation in sperm were observed in the sperm of mature males that had experienced early rearing in either a hatchery or stream environment. However, many fewer loci were analyzed for differential methylation in sperm as over 90% of the covered CG sites were filtered for constitutive hypermethylation across all samples (Table 1). The high level of methylation is a characteristic feature of vertebrate sperm DNA [69,99,100]. Despite the relatively small number of CG sites analyzed in sperm, we identified 19 DMCs and 8 DMRs. These loci were both hyper- and hypo-methylated, in roughly equal proportions, in sperm of hatchery- compared to stream-reared fish. The largest DMR in terms of number of CG involved as well as percent difference was associated with a *cyclic AMP-responsive element-binding protein* gene, a transcription factor with known function in spermatogenesis [101]. A single DMC, making up a larger DMR in sperm, was within 100 bp of a DMC identified in the liver of the age-2 males. This sperm DMR was within 5 kb of heat shock cognate 70, a non-inducible member of the heat shock protein 70 family. The remaining DMCs were unique to sperm. Recent studies focused on identifying mechanisms and biomarkers of sperm quality have identified specific DNA methylation signatures in low quality sperm in mammals [102] and in cultured striped bass [103]. While it would be speculative to comment on the functional significance of DNA methylation differences observed in this study, they provide interesting targets for future studies aimed at identifying relationships between DNA methylation, gene expression patterns and sperm related phenotypes in hatchery-reared steelhead.

Fewer DMCs and DMRs were identified in sperm in the current study compared to a previous study in Methow River steelhead that identified over 100 DMRs in sperm of wild-caught hatchery versus natural-origin steelhead [30]. The difference in the number of differentially methylated loci identified between these two studies could be at least partially attributable to differences in aspects of the study design. For example, males in the current study were generally 2–4 years younger than those of the previous study and regardless of rearing treatment, all males in the current study were spawned and reared for the first 2 mpf under hatchery conditions whereas natural-origin fish in the previous study were wild-spawned. Further, methodological differences between the studies make it difficult to directly compare overlaps in CG analyzed in both studies. However, despite the differences in samples and methodology, a single gene, *oocyte Zn finger XICOF20*, which encodes a zinc finger protein with a Krüppel-associated box (KRAB) domain, was found to be associated with DMCs in the sperm of hatchery-reared fish in both studies. Zinc finger proteins are transcription factors with functions related to DNA/RNA binding, RNA splicing, and protein-protein interactions [104]. Zinc finger proteins are found in high abundance in sperm, and although the function of these proteins in sperm is still controversial, it has been suggested that they could be important in paternal transmission of epigenetic information to offspring [105]. This gene provides an interesting target for future studies related to understanding the potential role of DNA methylation in fitness related phenotypes observed in hatchery-reared salmon. It is important to note that RRBS only considers a small portion of CG sites in the genome, and that additional differentially methylated regions may be identified through the use of whole-genome bisulfite sequencing approaches. In addition, intergenerational DNA methylation could also be passed through the egg and we focused solely on males for this study.

The effect of family on DNA methylation is the most notable result from global methylation analysis in sperm compared to liver. Hierarchical clustering showed very strong relationships between methylation patterns in the sperm of siblings compared to unrelated individuals (Figure 8). This effect of family on DNA methylation has also been reported in the gametes of oysters [106] and in one-month old whole stickleback [107]. This family effect is at least in part due to the underlying genetic sequence in or near specific CG loci [108,109,110]. The role of genetic variation in determining DNA methylation has the potential to confound or bias associations between DNA methylation and environmental variables if not properly accounted for, particularly if the effect size is small, which is often the case for environmental conditions of interest [15]. The genetic-epigenetic interaction is likely to be similar across somatic and germline cell-types [111], but we likely only observed it in sperm where the effect of rearing treatment was small. These results highlight the importance of controlling for genetic variation as it can lead to spurious associations between DNA methylation and environmental variables of interest [112]. In the current study, we removed potential CG SNPs from our analyses and also included kinship as a model covariate in our differential methylation analyses to try to minimize the effect of genotype on the differential methylation analysis. Our results suggest that future studies in natural populations should try to control for genetic effects on DNA methylation as much as possible to avoid potential confounding effects.

## 5. Conclusions

This study is the first to assess the effects of hatchery rearing on DNA methylation patterns in fish across developmental time and in multiple tissues. We found a strong effect of rearing environment on DNA methylation patterns in the liver of males at 10 mpf. While majority of the loci affected by the early rearing environment were reversible, the early-rearing environment continued to affect DNA methylation patterns in the liver after a change in environment (1 year of hatchery rearing). Direct comparison of the liver methylation patterns in age-1 versus age-2 fish highlight the plasticity of DNA methylation across developmental time. The largest differences in methylation were between the age-1 and age-2 fish and genes associated with these DMCs appear to at least in part reflect physiological requirements/changes for smoltification. Results in sperm show little effect of early-rearing on DNA methylation patterns, but strong family effects were observed, highlighting the importance in accounting for relatedness in experimental design. The results of this study are consistent with previous work that hatchery rearing does have impacts on DNA methylation, but highlight the fact that temporal and tissue-specific differences are important to understand for interpretation and implications for phenotypes associated with captive rearing practices.

## Figures and Tables

**Table 1:** Breakdown of number of CG dinucleotides filtered for each sample contrast. The final row, “CG remaining after filtering for CG single nucleotide polymorphisms (SNPs)”, represents the number of CG analyzed for differential methylation analysis for each sample contrast.

Sample Contrast	*Immediate*	*Persistent*	*Developmental*	*Intergenerational*
Tissue	liver	liver	liver	sperm
Sample size	20	19	39	60
CG covered across half of samples at > 10x coverage	1,449,887	1,251,548	1,354,166	1,494,268
CG remaining after filtering for low variation across samples	1,377,392	1,183,061	1,286,457	1,419,554
CG remaining after filtering for hypo-methylation	1,318,452	1,117,756	1,236,987	1,359,452
CG remaining after filtering for hyper-methylation	559,680	443,280	492,583	84,661
CG remaining after filtering for CG SNPs	532,017	418,800	465,391	67,661

**Table 2:** Top 30 most significantly enriched canonical pathways associated with genes that are differentially methylated in the liver of age-2 compared to age-1 males. Ratio column indicates number of affected genes over the total genes in the pathway.

Ingenuity Canonical Pathways	*P*-Value	Ratio
Axonal Guidance Signaling	6.31 × 10^−11^	266/421
CREB Signaling in Neurons	2.40 × 10^−10^	138/198
Role of NFAT in Cardiac Hypertrophy	3.55 × 10^−10^	137/197
G-Protein Coupled Receptor Signaling	1.23 × 10^−9^	169/255
GNRH Signaling	3.24 × 10^−8^	107/154
Netrin Signaling	5.75 × 10^−8^	49/60
Molecular Mechanisms of Cancer	7.08 × 10^−8^	217/352
Opioid Signaling Pathway	8.51 × 10^−8^	142/217
Neuropathic Pain Signaling in Dorsal Horn Neurons	1.48 × 10^−7^	80/111
PPARα/RXRα Activation	2.82 × 10^−7^	104/153
cAMP-mediated signaling	2.82 × 10^−7^	128/195
Synaptic Long-Term Depression	3.31 × 10^−7^	109/162
Adrenomedullin signaling pathway	6.46 × 10^−7^	119/181
nNOS Signaling in Skeletal Muscle Cells	1.00 × 10^−6^	32/37
GPCR-Mediated Nutrient Sensing in Enteroendocrine Cells	1.51 × 10^−6^	70/98
G Beta Gamma Signaling	1.70 × 10^−6^	77/110
Hepatic Fibrosis/Hepatic Stellate Cell Activation	1.70 × 10^−6^	95/141
Leukocyte Extravasation Signaling	2.00 × 10^−6^	113/173
Synaptic Long-Term Potentiation	2.34 × 10^−6^	79/114
Protein Kinase A Signaling	3.24 × 10^−6^	194/322
Dopamine-DARPP32 Feedback in cAMP Signaling	4.37 × 10^−6^	95/143
Regulation of the Epithelial-Mesenchymal Transition Pathway	5.89 × 10^−6^	116/181
PTEN Signaling	6.61 × 10^−6^	79/116
GABA Receptor Signaling	6.92 × 10^−6^	59/82
Corticotropin Releasing Hormone Signaling	6.92 × 10^−6^	87/130
Neuregulin Signaling	8.13 × 10^−6^	63/89
Signaling by Rho Family GTPases	8.51 × 10^−6^	137/220
Cellular Effects of Sildenafil (Viagra)	9.33 × 10^−6^	74/108
RAR Activation	1.05 × 10^−5^	104/161
Glutamate Receptor Signaling	1.12 × 10^−5^	39/50

**Table 3:** *Developmental* differentially methylated regions (DMRs) with the most extreme methylation differences (>40%). The methylation difference is relative to the age-1 samples, with negative methylation differences indicating a loss of methylation in the age-2 livers.

DMR_ID (chr.start.stop)	Number of CG	Methylation Difference	Relationship DMR to Gene	Gene
NC_035079.1.50470786.50470912	8	−60.7	0	plakophilin-4
NC_035086.1.43986683.43986897	13	−58.4	0	coagulation factor IX
NC_035090.1.20320098.20320139	3	−55.9	0	thyroid hormone receptor beta
NC_035079.1.54857805.54857941	7	−55.5	0	aryl hydrocarbon receptor
NC_035094.1.42211884.42211949	4	−53.9	0	thyroid hormone receptor beta
NC_035098.1.39974903.39974975	6	−51.9	−4743	activin receptor type-2A
NC_035103.1.6240302.6240419	8	−51.0	0	protein phosphatase 1 regulatory subunit 37
NC_035077.1.65859491.65859615	5	−48.6	0	tetratricopeptide repeat protein 27
NC_035104.1.2409783.2409794	3	−45.6	0	kinesin-1 heavy chain
NC_035092.1.14183000.14183073	3	−45.1	0	transcription factor 7-like 2
NC_035079.1.70446509.70446615	9	−44.6	0	B-cell CLL/lymphoma 9 protein
NW_018557253.1.30187.30270	6	−44.2	0	thrombospondin-2
NC_035096.1.18319228.18319305	5	−44.1	0	nuclear factor erythroid 2-related factor 1
NC_035089.1.22399625.22399721	6	−43.9	0	interferon regulatory factor 2-binding protein 2-B
NC_035084.1.6294684.6294737	3	−42.5	0	cholesterol 24-hydroxylase
NC_035080.1.55253415.55253602	3	−42.4	4013	5-hydroxytryptamine receptor 1E
NC_035078.1.62045061.62045113	3	−42.0	0	NLR family CARD domain-containing protein 3

## Supplementary Materials

The following are available online at https://www.mdpi.com/2073-4425/10/5/356/s1, Supplementary File 1: Figures S1–S3 and Tables S1–S4. Figure S1. Average daily water temperature in the hatchery tanks and stream channel during the experimental timeframe. Figure S2. Total count and proportion of immature and mature males at age-2 per family separated by rearing treatment. Figure S3. Hierarchical clustering of sperm DMCs from the Intergenerational time-point. Table S1. Enriched canonical pathways for Immediate Liver Samples. Table S2. Enriched canonical pathways for Persistent Liver Samples. Table S3. Overlapping DMC between Immediate and Persistent sample contrasts. Table S4. Enriched canonical pathways for Developmental liver samples. Supplementary File 2: Dataset S1. Sequencing information per sample in RRBS analysis. Supplementary File 3: Dataset S2. Body size and family assignment for individuals used in RRBS and family metadata. Supplementary File 4: Dataset S3. DMCs and DMRs for each of the four sample contrasts: Immediate, Persistent, Developmental and Intergenerational.
