# Supplementary material for: Temporal Dynamics of DNA Methylation Patterns in Response to Rearing Juvenile Steelhead (Oncorhynchus mykiss) in a Hatchery versus Simulated Stream Environment
Source: Genes (Basel). 2019 May 9;10(5):356. doi: 10.3390/genes10050356 (PMC6563097; doi:10.3390/genes10050356)
Supplement: Supplementary file 1 [file genes-10-00356-s001.zip › supplementary_revised/Supplementary_File1.pdf]

Figure S1. Average daily water temperature in the hatchery tanks and simulated stream during the experimental timeframe at Manchester. Prior to 8 August 2014, hatchery fish were reared at the WNFH.

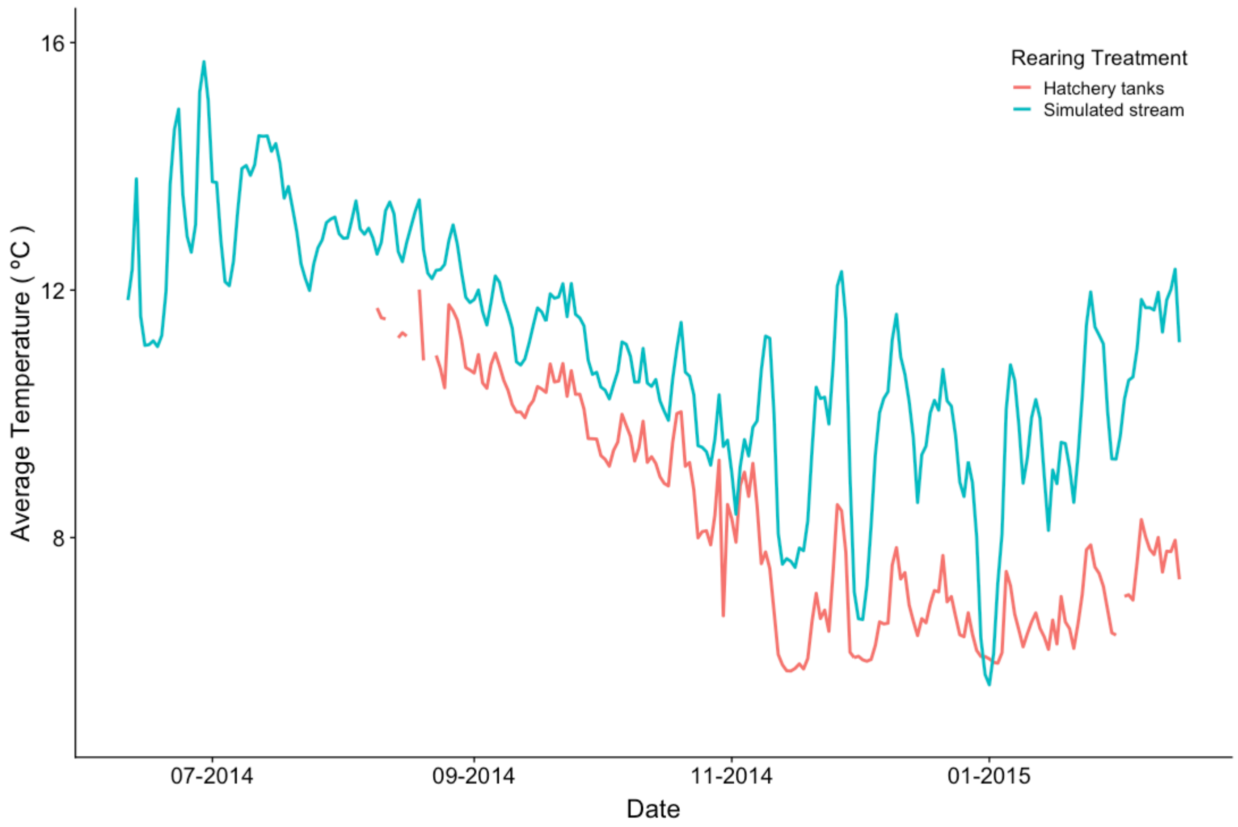

Figure S2. Total count and proportion of immature and mature males at age 2 per family separated by rearing treatment.

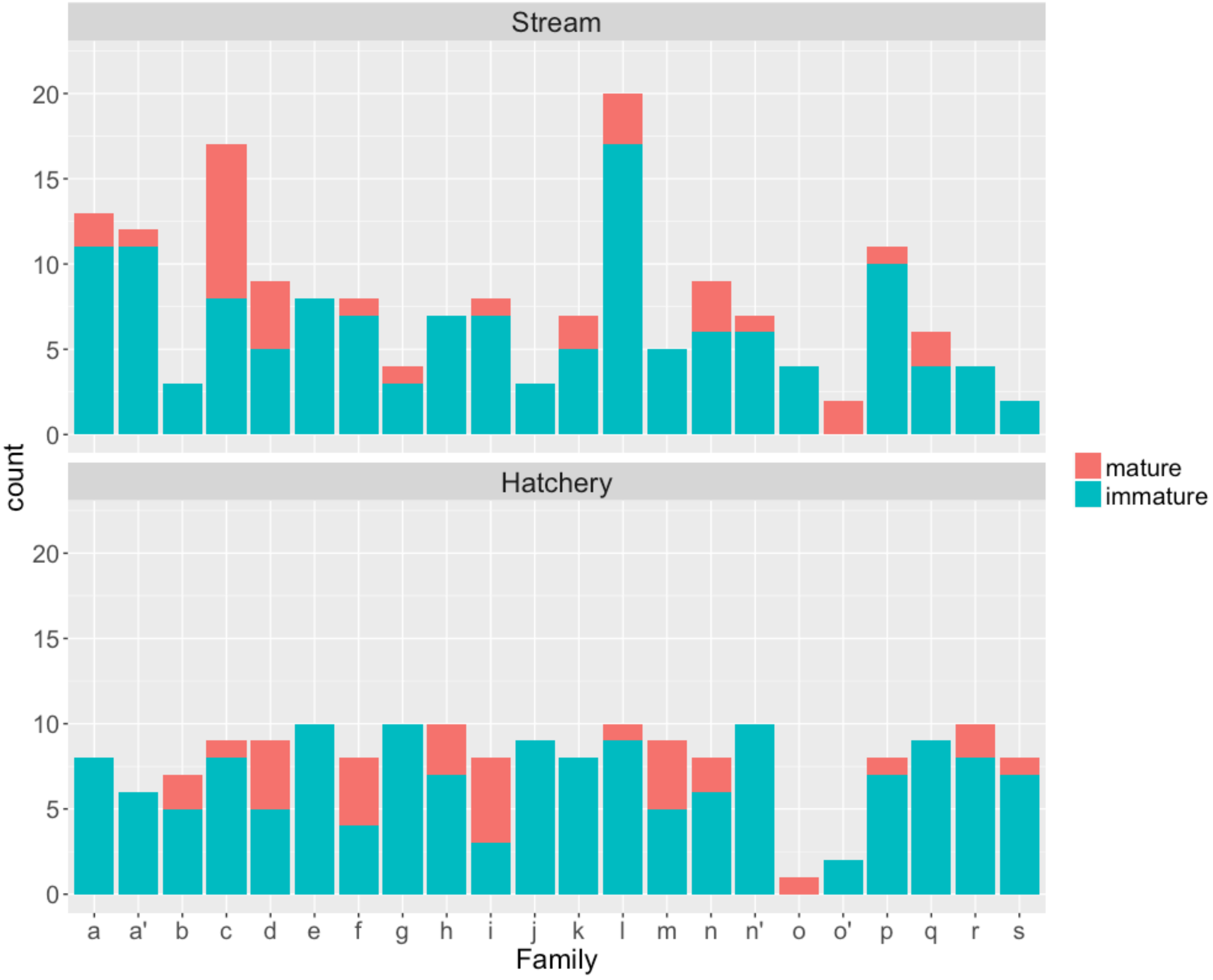

Figure S3. Hierarchical clustering of sperm DMCs from the Intergenerational time-point. The rearing-group is identified by color (hatchery = blue, stream = red) at the top of the column. Each row represents a DMC. The heatmap depicts percent methylation for each DMC for each individual with the darkest red indicating 100% methylation and the lightest indicating 0% methylation. The regions that did not meet the coverage cutoff for a particular individual are represented by gray boxes.

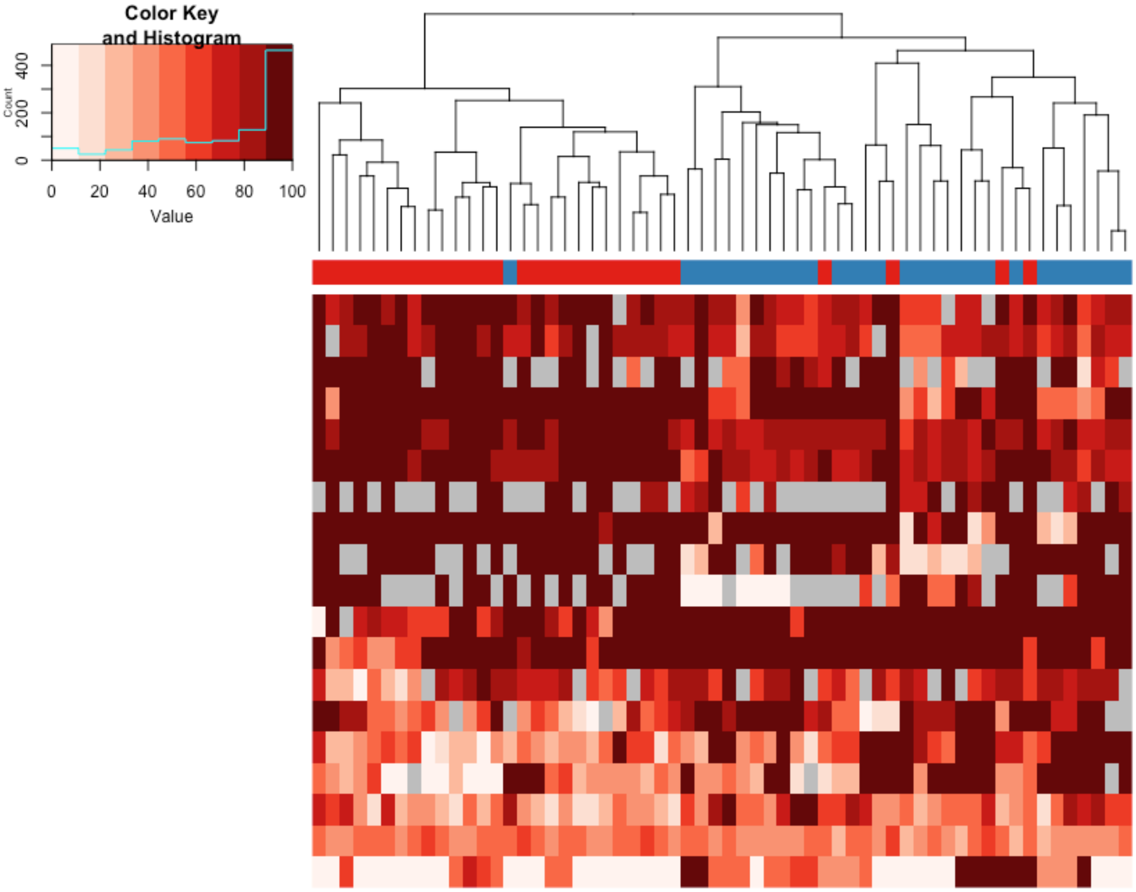

Table S1. Enriched canonical pathways for *Immediate* Liver Samples

Ingenuity Canonical Pathways	p-value	Number Molecules
VDR/RXR Activation	1.45E-04	7
Nitric Oxide Signaling in the Cardiovascular System	4.07E-03	7
CREB Signaling in Neurons	5.62E-03	10
Calcium-induced T Lymphocyte Apoptosis	6.31E-03	4
TR/RXR Activation	6.76E-03	6
Heparan Sulfate Biosynthesis (Late Stages)	1.05E-02	4
Role of NFAT in Cardiac Hypertrophy	1.55E-02	9
Heparan Sulfate Biosynthesis	1.74E-02	4
Cellular Effects of Sildenafil (Viagra)	1.95E-02	6
Calcium Signaling	2.04E-02	8
Neuropathic Pain Signaling In Dorsal Horn Neurons	2.19E-02	6
RhoGDI Signaling	2.29E-02	7
Dermatan Sulfate Biosynthesis (Late Stages)	2.40E-02	3
VEGF Family Ligand-Receptor Interactions	2.45E-02	5
Chondroitin Sulfate Biosynthesis (Late Stages)	2.63E-02	3
Notch Signaling	2.63E-02	3
tRNA Splicing	2.82E-02	3
Huntington's Disease Signaling	2.82E-02	9
G-Protein Coupled Receptor Signaling	2.88E-02	10
PPARα/RXRα Activation	3.09E-02	7
Ephrin B Signaling	3.47E-02	4
Epithelial Adherens Junction Signaling	3.72E-02	6
RAR Activation	3.89E-02	7
Hypoxia Signaling in the Cardiovascular System	3.98E-02	4
Role of Oct4 in Mammalian Embryonic Stem Cell Pluripotency	4.27E-02	3
Chondroitin Sulfate Biosynthesis	4.57E-02	3
EGF Signaling	4.57E-02	4
Sperm Motility	4.68E-02	5
Gustation Pathway	5.01E-02	5
Fc Epsilon RI Signaling	5.13E-02	5
Dermatan Sulfate Biosynthesis	5.50E-02	3

Table S2. Enriched canonical pathways for *Persistent* Liver Samples

**Ingenuity Canonical Pathways**	**p-value**	**Number molecules**
CREB Signaling in Neurons	2.88E-03	10
PI3K Signaling in B Lymphocytes	4.47E-03	7
Synaptic Long Term Depression	8.51E-03	8
Axonal Guidance Signaling	8.51E-03	15
Chronic Myeloid Leukemia Signaling	1.12E-02	6
Glutamate Receptor Signaling	1.20E-02	4
Calcium Signaling	1.23E-02	8
Neuropathic Pain Signaling In Dorsal Horn Neurons	1.45E-02	6
GABA Receptor Signaling	1.55E-02	5
Melatonin Signaling	2.57E-02	4
Gα12/13 Signaling	2.95E-02	6
Semaphorin Signaling in Neurons	4.07E-02	3
Bladder Cancer Signaling	5.25E-02	4
Synaptic Long Term Potentiation	5.25E-02	5

Table S3. Overlapping DMC between *Immediate* and *Persistent* sample contrasts

Persistent_DMC_ID	Immediate_DMC_ID	Distance Between DMCs (bp)	geneID	geneID	gene_desc	meth_diff_ persistent	meth_diff_ immediate
NC_035090.1.51479257	NC_035090.1.51475970	3286	gene26690	Q13474.2	Dystrophin-related protein 2	-33.6	-22.9
NC_035078.1.72178978	NC_035078.1.72176787	2190	.	.	.	25	-13.6
NC_035079.1	NC_035079.1.37901381	1121	gene5142	P17247.1	Transforming growth factor beta-2	9.9	-21.3
NC_035100.1	NC_035100.1.16398197	217	.	.	.	18.4	-25.7
NC_035101.1	NC_035101.1.25373589	60	.	.	.	-36.6	37.9
NC_035104.1.20809908	NC_035104.1.20809908	0	.	.	.	19.7	-17.1
NC_035083.1.30499549	NC_035083.1.30506977	7427	gene12587	Q8QHJ9.2	Interleukin-17 receptor D	-31.6	17.4

Table S4. Enriched canonical pathways for Developmental Liver Samples

Ingenuity Canonical Pathways	p-value	Number Molecules
Axonal Guidance Signaling	6.31E-11	266
CREB Signaling in Neurons	2.40E-10	138
Role of NFAT in Cardiac Hypertrophy	3.55E-10	137
G-Protein Coupled Receptor Signaling	1.23E-09	169
GNRH Signaling	3.24E-08	107
Netrin Signaling	5.75E-08	49
Molecular Mechanisms of Cancer	7.08E-08	217
Opioid Signaling Pathway	8.51E-08	142
Neuropathic Pain Signaling In Dorsal Horn Neurons	1.48E-07	80
PPARα/RXRα Activation	2.82E-07	104
cAMP-mediated signaling	2.82E-07	128
Synaptic Long Term Depression	3.31E-07	109
Adrenomedullin signaling pathway	6.46E-07	119
nNOS Signaling in Skeletal Muscle Cells	1.00E-06	32
GPCR-Mediated Nutrient Sensing in Enteroendocrine Cells	1.51E-06	70
G Beta Gamma Signaling	1.70E-06	77
Hepatic Fibrosis / Hepatic Stellate Cell Activation	1.70E-06	95
Leukocyte Extravasation Signaling	2.00E-06	113
Synaptic Long Term Potentiation	2.34E-06	79
Protein Kinase A Signaling	3.24E-06	194
Dopamine-DARPP32 Feedback in cAMP Signaling	4.37E-06	95
Regulation of the Epithelial-Mesenchymal Transition Pathway	5.89E-06	116
PTEN Signaling	6.61E-06	79
GABA Receptor Signaling	6.92E-06	59
Corticotropin Releasing Hormone Signaling	6.92E-06	87
Neuregulin Signaling	8.13E-06	63
Signaling by Rho Family GTPases	8.51E-06	137
Cellular Effects of Sildenafil (Viagra)	9.33E-06	74
RAR Activation	1.05E-05	104
Glutamate Receptor Signaling	1.12E-05	39
GP6 Signaling Pathway	1.12E-05	83
IGF-1 Signaling	1.17E-05	72
Colorectal Cancer Metastasis Signaling	1.17E-05	137
Tight Junction Signaling	1.32E-05	93
Factors Promoting Cardiogenesis in Vertebrates	1.58E-05	57
p70S6K Signaling	1.78E-05	83
IL-7 Signaling Pathway	2.00E-05	55
ErbB Signaling	2.14E-05	69
Cardiac Hypertrophy Signaling	2.14E-05	130

Nitric Oxide Signaling in the Cardiovascular System	2.45E-05	70
Calcium Signaling	3.31E-05	109
Gustation Pathway	3.55E-05	69
Melatonin Signaling	3.80E-05	46
Ephrin Receptor Signaling	3.89E-05	101
Role of Osteoblasts, Osteoclasts and Chondrocytes in Rheumatoid Arthritis	3.89E-05	117
Gap Junction Signaling	4.57E-05	114
Gαi Signaling	5.13E-05	72
Type II Diabetes Mellitus Signaling	5.25E-05	90
Neuroinflammation Signaling Pathway	5.75E-05	137
Epithelial Adherens Junction Signaling	5.89E-05	82
Human Embryonic Stem Cell Pluripotency	7.08E-05	84
IL-8 Signaling	7.08E-05	110
Sperm Motility	7.24E-05	67
Apelin Cardiomyocyte Signaling Pathway	7.24E-05	71
FGF Signaling	7.94E-05	60
Agrin Interactions at Neuromuscular Junction	8.91E-05	47
Amyotrophic Lateral Sclerosis Signaling	1.02E-04	70
Role of Macrophages, Fibroblasts and Endothelial Cells in Rheumatoid Arthritis	1.26E-04	150
Renin-Angiotensin Signaling	1.32E-04	77
Wnt/β-catenin Signaling	1.45E-04	88
CCR5 Signaling in Macrophages	1.51E-04	50
Endothelin-1 Signaling	1.70E-04	108
Ephrin A Signaling	1.78E-04	41
Apelin Endothelial Signaling Pathway	1.82E-04	76
B Cell Receptor Signaling	2.24E-04	101
Melanocyte Development and Pigmentation Signaling	2.29E-04	65
Rac Signaling	2.45E-04	75
P2Y Purigenic Receptor Signaling Pathway	2.45E-04	80
STAT3 Pathway	2.88E-04	72
Cardiac β-adrenergic Signaling	2.88E-04	72
FcγRIIB Signaling in B Lymphocytes	3.02E-04	55
Osteoarthritis Pathway	3.24E-04	102
Sertoli Cell-Sertoli Cell Junction Signaling	3.31E-04	96
Ceramide Signaling	3.72E-04	61
ERK/MAPK Signaling	3.80E-04	105
Endocannabinoid Developing Neuron Pathway	3.89E-04	76
Thrombin Signaling	4.27E-04	115
RhoGDI Signaling	4.37E-04	89
PI3K Signaling in B Lymphocytes	4.57E-04	73
iCOS-iCOSL Signaling in T Helper Cells	4.68E-04	59
EGF Signaling	4.79E-04	47
Gαs Signaling	5.13E-04	60

IL-3 Signaling	5.37E-04	56
FAK Signaling	5.37E-04	65
VDR/RXR Activation	5.75E-04	41
PI3K/AKT Signaling	5.75E-04	71
PKCθ Signaling in T Lymphocytes	6.17E-04	83
Non-Small Cell Lung Cancer Signaling	6.76E-04	54
VEGF Signaling	7.24E-04	64
BMP signaling pathway	7.76E-04	47
Androgen Signaling	8.32E-04	71
Actin Cytoskeleton Signaling	8.32E-04	119
Leptin Signaling in Obesity	9.55E-04	53
GPCR-Mediated Integration of Enteroendocrine Signaling Exemplified by an L Cell	9.77E-04	41
Gαq Signaling	9.77E-04	86
Role of NANOG in Mammalian Embryonic Stem Cell Pluripotency	1.00E-03	74
Germ Cell-Sertoli Cell Junction Signaling	1.07E-03	95
Th1 and Th2 Activation Pathway	1.12E-03	70
AMPK Signaling	1.23E-03	115
Integrin Signaling	1.23E-03	115
GDNF Family Ligand-Receptor Interactions	1.32E-03	52
Insulin Receptor Signaling	1.32E-03	79
Fcγ Receptor-mediated Phagocytosis in Macrophages and Monocytes	1.45E-03	53
Acute Myeloid Leukemia Signaling	1.48E-03	58
Paxillin Signaling	1.48E-03	69
HIPPO signaling	1.55E-03	49
HGF Signaling	1.58E-03	70
Pancreatic Adenocarcinoma Signaling	1.66E-03	71
TR/RXR Activation	1.70E-03	55
CXCR4 Signaling	1.70E-03	92
Role of Tissue Factor in Cancer	1.74E-03	72
Phagosome Formation	1.95E-03	62
Dendritic Cell Maturation	1.95E-03	74
ErbB2-ErbB3 Signaling	2.14E-03	48
HER-2 Signaling in Breast Cancer	2.14E-03	58
TGF-β Signaling	2.19E-03	53
T Cell Exhaustion Signaling Pathway	2.24E-03	77
CDK5 Signaling	2.29E-03	59
Virus Entry via Endocytic Pathways	2.34E-03	65
Circadian Rhythm Signaling	2.45E-03	23
UVA-Induced MAPK Signaling	2.45E-03	66
Gα12/13 Signaling	2.45E-03	79
Erythropoietin Signaling	2.51E-03	55
ILK Signaling	2.57E-03	96
Role of Wnt/GSK-3β Signaling in the Pathogenesis of Influenza	2.63E-03	37

Reelin Signaling in Neurons	2.69E-03	56
Angiopoietin Signaling	2.75E-03	51
Basal Cell Carcinoma Signaling	2.88E-03	42
Breast Cancer Regulation by Stathmin1	2.88E-03	110
Relaxin Signaling	3.16E-03	86
ErbB4 Signaling	3.24E-03	48
IL-12 Signaling and Production in Macrophages	3.24E-03	65
Mouse Embryonic Stem Cell Pluripotency	3.24E-03	65
CCR3 Signaling in Eosinophils	3.55E-03	67
FAT10 Cancer Signaling Pathway	3.63E-03	28
Apelin Pancreas Signaling Pathway	3.80E-03	36
IL-17A Signaling in Airway Cells	3.80E-03	45
PAK Signaling	3.80E-03	62
fMLP Signaling in Neutrophils	3.89E-03	69
14-3-3-mediated Signaling	3.89E-03	76
UVB-Induced MAPK Signaling	4.07E-03	41
NGF Signaling	4.27E-03	71
Wnt/Ca+ pathway	4.68E-03	38
IL-6 Signaling	4.68E-03	66
Dermatan Sulfate Biosynthesis	4.79E-03	30
Dermatan Sulfate Biosynthesis (Late Stages)	4.90E-03	23
Endocannabinoid Cancer Inhibition Pathway	5.01E-03	84
NF-κB Activation by Viruses	5.13E-03	55
PXR/RXR Activation	5.50E-03	31
FLT3 Signaling in Hematopoietic Progenitor Cells	5.75E-03	51
Th2 Pathway	5.75E-03	57
T Cell Receptor Signaling	6.03E-03	58
HMGB1 Signaling	6.03E-03	65
Sonic Hedgehog Signaling	6.17E-03	18
Chondroitin Sulfate Biosynthesis	6.17E-03	28
IL-15 Signaling	6.46E-03	47
IL-4 Signaling	6.76E-03	48
JAK/Stat Signaling	6.76E-03	54
Phototransduction Pathway	7.08E-03	29
Neurotrophin/TRK Signaling	7.24E-03	49
Chronic Myeloid Leukemia Signaling	7.41E-03	63
Ovarian Cancer Signaling	7.76E-03	81
Glioblastoma Multiforme Signaling	7.94E-03	92
CNTF Signaling	8.32E-03	40
Xenobiotic Metabolism Signaling	8.51E-03	124
Cholecystokinin/Gastrin-mediated Signaling	8.91E-03	53
Prolactin Signaling	8.91E-03	53
Macropinocytosis Signaling	8.91E-03	53
Hepatic Cholestasis	8.91E-03	68

LPS-stimulated MAPK Signaling	9.33E-03	54
α-Adrenergic Signaling	9.55E-03	48
Production of Nitric Oxide and Reactive Oxygen Species in Macrophages	9.77E-03	92
Regulation of IL-2 Expression in Activated and Anergic T Lymphocytes	1.02E-02	43
Endometrial Cancer Signaling	1.07E-02	44
p38 MAPK Signaling	1.07E-02	50
VEGF Family Ligand-Receptor Interactions	1.12E-02	51
Myc Mediated Apoptosis Signaling	1.15E-02	45
Chemokine Signaling	1.20E-02	40
GM-CSF Signaling	1.20E-02	46
IL-15 Production	1.23E-02	15
Interferon Signaling	1.23E-02	15
Role of NFAT in Regulation of the Immune Response	1.26E-02	82
HIF1α Signaling	1.29E-02	63
Clathrin-mediated Endocytosis Signaling	1.29E-02	95
Docosahexaenoic Acid (DHA) Signaling	1.38E-02	31
Renal Cell Carcinoma Signaling	1.38E-02	49
Th1 Pathway	1.38E-02	49
Prostate Cancer Signaling	1.41E-02	57
eNOS Signaling	1.41E-02	87
SPINK1 General Cancer Pathway	1.45E-02	37
Natural Killer Cell Signaling	1.45E-02	58
RhoA Signaling	1.55E-02	60
Amyloid Processing	1.62E-02	28
Cancer Drug Resistance By Drug Efflux	1.62E-02	28
Ephrin B Signaling	1.62E-02	39
Granulocyte Adhesion and Diapedesis	1.70E-02	54
IL-17 Signaling	1.74E-02	47
Growth Hormone Signaling	1.82E-02	48
RANK Signaling in Osteoclasts	1.86E-02	57
Glioma Signaling	1.86E-02	67
UVC-Induced MAPK Signaling	1.91E-02	30
Inhibition of Angiogenesis by TSP1	1.95E-02	21
CD28 Signaling in T Helper Cells	1.95E-02	59
Role of IL-17A in Arthritis	2.04E-02	37
Chondroitin Sulfate Biosynthesis (Late Stages)	2.19E-02	22
Heparan Sulfate Biosynthesis	2.19E-02	32
nNOS Signaling in Neurons	2.24E-02	27
Dopamine Receptor Signaling	2.29E-02	39
MSP-RON Signaling Pathway	2.34E-02	33
Role of JAK family kinases in IL-6-type Cytokine Signaling	2.45E-02	15
Apelin Cardiac Fibroblast Signaling Pathway	2.51E-02	12
Role of MAPK Signaling in the Pathogenesis of Influenza	2.57E-02	35

PCP pathway	2.57E-02	35
Thrombopoietin Signaling	2.57E-02	42
Prostanoid Biosynthesis	2.63E-02	7
Glucocorticoid Receptor Signaling	2.88E-02	127
PDGF Signaling	2.95E-02	55
Fc Epsilon RI Signaling	3.16E-02	59
IL-2 Signaling	3.24E-02	40
Small Cell Lung Cancer Signaling	3.24E-02	49
D-myo-inositol (1,4,5)-Trisphosphate Biosynthesis	3.31E-02	17
Semaphorin Signaling in Neurons	3.31E-02	27
Remodeling of Epithelial Adherens Junctions	3.31E-02	33
Sphingosine-1-phosphate Signaling	3.47E-02	64
Tec Kinase Signaling	3.47E-02	78
Phospholipase C Signaling	3.47E-02	100
Eicosanoid Signaling	3.55E-02	28
PEDF Signaling	3.55E-02	53
Role of PI3K/AKT Signaling in the Pathogenesis of Influenza	3.63E-02	35
Melanoma Signaling	3.72E-02	36
TREM1 Signaling	3.80E-02	29
SAPK/JNK Signaling	3.89E-02	57
Calcium-induced T Lymphocyte Apoptosis	3.98E-02	24
PPAR Signaling	3.98E-02	47
Role of JAK1, JAK2 and TYK2 in Interferon Signaling	4.07E-02	11
Role of JAK2 in Hormone-like Cytokine Signaling	4.07E-02	19
Superpathway of Inositol Phosphate Compounds	4.27E-02	109
Regulation of Cellular Mechanics by Calpain Protease	4.68E-02	34
Notch Signaling	4.79E-02	21
Heparan Sulfate Biosynthesis (Late Stages)	4.79E-02	27
Agranulocyte Adhesion and Diapedesis	4.79E-02	56
Antioxidant Action of Vitamin C	5.13E-02	47
NF-κB Signaling	5.13E-02	83
